# Integration of family planning services into HIV care clinics: Results one year after a cluster randomized controlled trial in Kenya

**DOI:** 10.1371/journal.pone.0172992

**Published:** 2017-03-22

**Authors:** Craig R. Cohen, Daniel Grossman, Maricianah Onono, Cinthia Blat, Sara J. Newmann, Rachel L. Burger, Starley B. Shade, Norah Bett, Elizabeth A. Bukusi

**Affiliations:** 1 Department of Obstetrics, Gynecology & Reproductive Sciences, University of California San Francisco, San Francisco, California, United States of America; 2 Ibis Reproductive Health, Oakland, California, United States of America; 3 Centre for Microbiology Research, Kenya Medical Research Institute, Nairobi, Kenya; 4 Department of Medicine, University of California San Francisco, San Francisco, California, United States of America; 5 Department of Reproductive Health, Ministry of Health, Kisumu, Kenya; Public Library of Science, FRANCE

## Abstract

**Objectives:**

To determine if integration of family planning (FP) and HIV services led to increased use of more effective contraception (i.e. hormonal and permanent methods, and intrauterine devices) and decreased pregnancy rates.

**Design:**

Cohort analysis following cluster randomized trial, when the Kenya Ministry of Health led integration of the remaining control (delayed integration) sites and oversaw integrated services at the original intervention (early integration) sites.

**Setting:**

Eighteen health facilities in Kenya.

**Subjects:**

Women aged 18–45 receiving care: 5682 encounters at baseline, and 11628 encounters during the fourth quarter of year 2.

**Intervention:**

“One-stop shop” approach to integrating FP and HIV services.

**Main outcome measures:**

Use of more effective contraceptive methods and incident pregnancy across two years of follow-up.

**Results:**

Following integration of FP and HIV services at the six delayed integration clinics, use of more effective contraception increased from 31.7% to 44.2% of encounters (+12.5%; Prevalence ratio (PR) = 1.39 (1.19–1.63). Among the twelve early integration sites, the proportion of encounters at which women used more effective contraceptive methods was sustained from the end of the first to the second year of follow-up (37.5% vs. 37.0%). Pregnancy incidence including all 18 integrated sites in year two declined in comparison to the control arm in year one (rate ratio: 0.72; 95% CI 0.60–0.87).

**Conclusions:**

Integration of FP services into HIV clinics led to a sustained increase in the use of more effective contraceptives and decrease in pregnancy incidence 24 months following implementation of the integrated service model.

**Trial registration:**

ClinicalTrials.gov NCT01001507

## Introduction

Improving access to family planning is crucial to help the 16 million women living with HIV in sub-Saharan Africa achieve their fertility intentions and reduce vertical transmission of HIV.[1,2] Among the HIV-infected women living in this region, studies indicate that 62–93% of pregnancies are unintended.[3,4,5] The prevention of unintended pregnancy serves as an important component of a comprehensive prevention of mother-to-child transmission (PMTCT) strategy.[6] Greater use of family planning among HIV-infected women should also lead to a decrease in maternal morbidity and mortality, as well as poor neonatal outcomes including preterm birth.[7,8]

Strengthening HIV and reproductive health service integration is one of the ten goals set forward in the 2013 UNAIDS Report on the global AIDS epidemic.[9] While some countries have made progress towards integration of HIV and family planning services,[9] in many settings in sub-Saharan Africa, contraceptive services for HIV-infected individuals are only provided in family planning clinics rather than at clinics providing HIV care and treatment.[10] In recognition of the structural barriers associated with a referral model of care, multiple international statements have recommended integrating family planning and HIV services.[11] We recently reported findings from a cluster randomized trial (CRT) of a “one-stop shop” approach to integrating family planning services into the HIV care and treatment clinics in rural Kenya.[12] We found that integration was associated with 81% (95% CI 24% – 163%) greater odds of using more effective contraceptive methods (i.e. sterilization, intrauterine device (IUD), sub-dermal implants, injectables and oral contraceptives) with a non-significant decrease in condom use in comparison to patients receiving care at control facilities after 12 months of observation. Although no significant reduction in pregnancy incidence was observed during the study, one year may have been too short a period of observation to impact this outcome.[12] Furthermore, we found that integration of family planning into HIV services was acceptable to patients and providers,[13,14,15,16,17,18] inexpensive to implement and cost-efficient in the Kenyan setting.[19]

This cohort analysis was conducted one year after completion of the CRT. We sought to determine if the increased use of more effective contraceptives was sustainable and reproducible when implemented and managed by the Kenyan Ministry of Health during the second year of observation, and to determine if pregnancy incidence over 24 months of observation was affected by integration of family planning services.

## Methods

As described previously, we conducted a CRT at 18 public HIV care & treatment clinics in Nyanza Province, Kenya [12]. All sites were supported by Family AIDS Care & Education Services (FACES), a collaboration between University of California San Francisco (UCSF) and the Kenyan Medical Research Institute (KEMRI) supported by the Centers for Disease Control/President’s Emergency Plan for AIDS Relief (PEPFAR).

**Fig 1:**
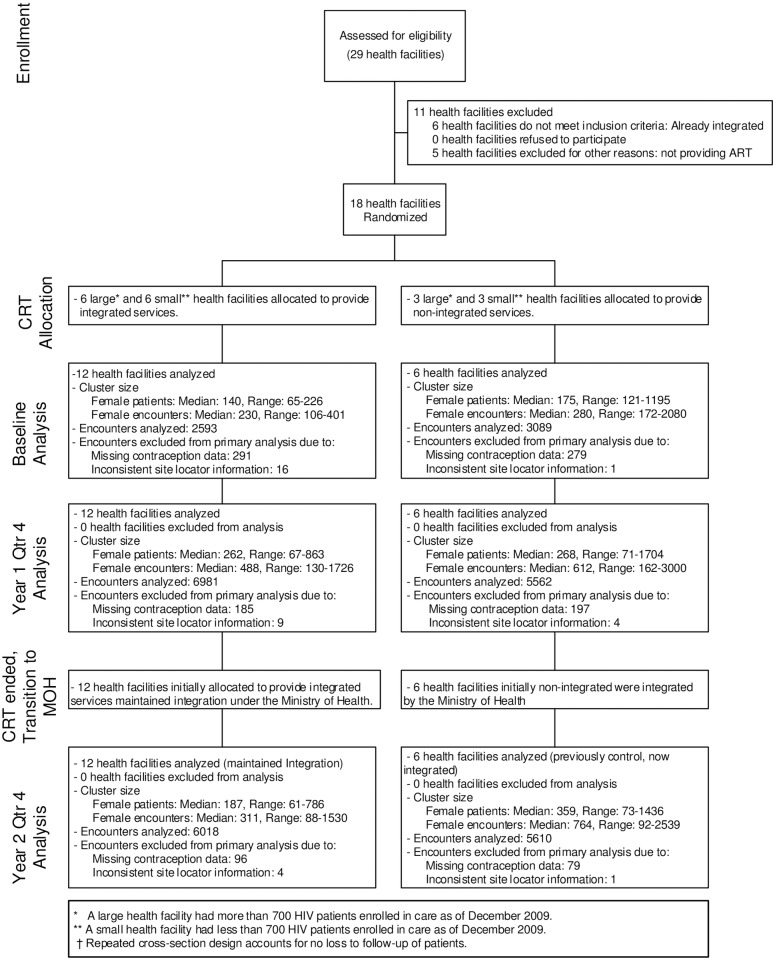
Trial profile.

Briefly, in preparation for the study, starting in March 2010, peer educators at all sites were trained to conduct group educational health talks about family planning to clients waiting to be seen at the HIV clinics. These health talks focused on why some HIV-infected individuals choose to use family planning and reviewed all available contraceptive methods, including their effectiveness and common side effects. Staff at the 18 study sites underwent training between June and August 2010. At HIV clinics assigned to be control sites, staff continued the standard practice of referring clients interested in receiving non-condom family planning to a separate maternal-child health/family planning clinic at the same facility. HIV clinics assigned to the intervention provided family planning counseling and provision into the HIV clinic according to guidelines established by the Kenyan Government. In addition to asking about interest in using family planning, HIV clinic staff at integrated sites also provided all reversible family planning methods within the HIV clinic. At control sites, all reversible family planning methods were available at the Maternal Child Health-Family Planning (MCH-FP) clinic, but family planning services were generally available at different times than the HIV clinic, often requiring women to wait or return at another time. Between October 2010 and September 2011, twelve sites were randomized to integrate family planning services into the HIV clinic following Kenyan Ministry of Health guidelines while six clinics served as controls where HIV-infected clients desiring contraception were referred to family planning clinics at the same facility (Fig 1). During the period of the CRT, the study’s research nurses and FACES staff provided ongoing support to study sites, including leading refresher training as needed and troubleshooting service-delivery problems. In November 2011, after the CRT ended, family planning services were integrated into the HIV clinic at the six control sites. This transition, including training, was conducted by Kenyan Ministry of Health reproductive health coordinators assisted by the study’s research nurses, and included a three-day contraceptive technology training, two-day practicum of counseling and method insertion, and provision of logistical support to rearrange the facilities to provide the integrated services. After November 2011, Ministry of Health staff provided supervision of the integrated HIV clinics at all 18 sites with minimal support by FACES staff.

For this study, we observed sites over three time periods: (1) a three-month period prior to integration (December 2009 –February 2010 [baseline]); (2) a one-year period when the 12 original intervention (early integration) sites were integrated under the CRT while the six control sites continued to link women attending the HIV support center to separate family planning services within the health facility (October 2010 –September 2011); and (3) an 11-month period after the CRT ended when the Ministry of Health integrated the original control (delayed integration) sites and oversaw a second year of integrated services at the early integration sites (December 2011 –October 2012). October—November 2011 were used to transition support for integrated services from the CRT research team to the Ministry of Health and thus were not included in the initial CRT or post-CRT evaluation periods (Fig 1). Family planning use during the last three-month period of each year of follow-up was compared to highlight trends over time. Women aged 18–45 receiving care at participating HIV clinics contributed data for the analysis: 2593 and 3089 clinical encounters, respectively, from the 12 early and six delayed integration clinics at baseline; 6981 and 5562 clinical encounters from the early and delayed integration clinics during the fourth quarter of year 1 (Y1Q4), respectively; and 6018 and 5610 encounters from the early and delayed integration clinics during the fourth quarter of year 2 (Y2Q4)(Fig 1).

Participant family planning method and pregnancy information were extracted from patient encounter data collected in OpenMRS, an open source electronic medical records (EMR) platform maintained by the FACES program. At eleven of the facilities, paper clinical encounter forms for all patients in care were routinely entered into OpenMRS. Data collection into OpenMRS was not routine at the remaining 7 sites and instead, to facilitate this evaluation, encounter forms for the first 50 female patients seen per month who met study age eligibility criteria and had contraceptive method and pregnancy clearly documented were retrospectively entered into OpenMRS [12].

The Committee on Human Research at UCSF (initial approval date August 7, 2007) and the Ethical Review Committee at KEMRI (initial approval date October 24, 2007) approved the program evaluation protocol enabling analysis of deidentified and delinked programmatic data. Thus, individual consent was not required. The study was registered on ClinTrials.gov (NCT01001507) October 22, 2009, with enrollment of the baseline period starting December 2009. The authors confirm that all ongoing and related trials for this intervention are registered.

### Measures

The main outcome measure was reported use of more effective contraceptive methods (sterilization, intrauterine device [IUD], implant, injectable, or oral contraceptives). Secondary outcomes were:

- Use of less effective family planning methods (barrier methods, including condoms used alone, spermicide, emergency contraception, or natural family planning),
- Use of any family planning method,
- Use of condoms, either alone or with another more effective method,
- Use of condoms with a more effect method of family planning (dual use), and
- Incident pregnancy rates

We attempted to collect data on whether pregnancy was intended but completion was too poor to permit use of this data. Contraceptive method use was ascertained at each visit. Encounter forms initially included fields for current method of contraception in use by the patient or partner, medications currently used or prescribed on day of visit (which included contraception), and frequency of condom use in the past month (only condom use reported as “all of the time” was considered use). In May 2012, FACES sites transitioned to the Kenya Ministry of Health 257 ‘Blue Card’, which collected methods currently in use by self or partner or prescribed on day of visit. Methods identified through any of these fields were ascribed to the patient on that visit. When a woman reported multiple methods, we applied a hierarchy for assignment of contraceptive method of more effective family planning use superseding less effective family planning use superseding no family planning use.

Pregnancy was diagnosed clinically through self-report or gravid presentation. We estimated the date of conception (DoC) from the earliest clinical record of pregnancy on which either the LMP or EDD was recorded and used this date to determine whether the pregnancy had occurred during the first or second year of follow-up. Methods for calculating the DoC have been previously described.[12] Due to resource constraints on data collection, pregnancies conceived during the second year of follow-up could only be detected through the close of the second year. This resulted in a relatively truncated detection window compared to pregnancies conceived in the first year, which could be first detected during the second year of observation. To avoid biases in comparisons across the two time periods we chose to artificially restrict detection of first year pregnancies to those documented on clinic visits in the first year.

### Statistical analysis

A series of log-binomial generalized estimating equations (GEE) models were built to calculate the prevalence of family planning method use at cross-sections over the study period and to compute the prevalence ratio for Y2Q4 compared to Y1Q4. Models were fitted with robust standard errors to account for clustering of encounters within site. Separate models were constructed for the outcomes: use of a more effective method vs. a less effective contraceptive method, or no method; use of any method vs. no method; use of a less effective method vs. a more effective method, or no method; use of condoms with a more effect method (dual use); and use of individual more effective methods or condoms. Each model included independent (predictor) variables: type of site (early- or delayed integration), time (baseline and two- to three-month categorizations of years one and two), and the interaction of type of site by time. Prevalence estimates were derived from model-based expected probabilities of method use rather than raw frequencies in order to correctly account for site-level clustering. Prevalence ratios and 95% confidence intervals for the prevalence in Y2Q4 compared to Y1Q4 were obtained through post-hoc contrasts of model coefficients. All prevalence estimates, prevalence ratios, and 95% confidence intervals were calculated from imputed data. To fill in gaps in family planning method documentation within the clinical record, during data management we carried over information about permanent and long-acting methods between visits. Specifically, a woman was assumed to be using an IUD or implant on any visit between two intervening reports of use, and was assumed sterilized on all visits subsequent to initial report. We tested the sensitivity of our findings to these assumptions by repeating the analysis of our primary outcome, use of a more effective contraceptive method: 1) assuming sterilization on all visits subsequent to initial report and assuming IUD or implant use on every visit after first report if there were two or more reports of use; and 2) without imputations.

We built three GEE models to test the effects of integration on pregnancy rates. The first model was used to estimate approximate pregnancy rates per 100 person-years of follow-up. Typically, Poisson regression would be applied to model an outcome as a rate. However, descriptive analyses of our pregnancy data found the variance of the incident pregnancy count substantially exceeded the mean, indicating a violation of the assumption of equal mean and variance underlying the Poisson model. Instead, we chose to use the negative binomial model, an extension of the Poisson which includes a scaling factor to correct for inequality of the mean and variance. The dependent variable was a count of incident pregnancies per site. Predictor terms were: type of site (early or delayed integration), follow-up year (year one or two), and a type of site by year interaction term. Sites contributed an observation for each year of follow-up, for a total of 36 observations. Robust standard errors were used to account for repeated measures per site. To obtain rates per 100 person-years of follow-up, the model included an offset term for the log of eligible women’s encounters over the year of follow-up multiplied by the mean interval between encounters (0.34 years) among women seen at sites where all encounters were entered into OpenMRS. The mean visit interval per encounter at the sites with complete data collection gives an approximate estimate of the at-risk period associated with each encounter at sites where sampling-based data collection does not allow for direct estimation of the at-risk period per patient. Encounters when a woman was already pregnant or could not become pregnant (<3 months after the EDD) were excluded from the offset.

The second model was constructed to provide a summary measure of the effect of integration on women’s pregnancy rates over the two years of follow-up. This model was identical to the first except that it included only one predictor term, integration status, which was coded as 1 if a site was integrated and 0 if a site was not integrated. For early integration sites, the integration status was the same in both years. For delayed sites, the integration status crossed over from not integrated in year one to integrated in year two. We took the exponent of the coefficient for integration status to obtain a rate ratio reflecting the pregnancy rates in years one and two among the early integrated sites and year two among the delayed integrated sites (numerator) over the pregnancy rate for the delayed sites in year one (denominator).

This third model compared the pregnancy rate across all sites in year two with the pregnancy rate in year one among the delayed integrated sites. Modeling methods were the same as those used for the second model except that early integration sites only contributed observations in year two. The purpose of this model was to test whether pregnancy rates differed when integration was carried out under the direction of the Kenyan Ministry of Health.

Descriptive analyses found substantial numbers of eligible women’s encounters were missing pregnancy status (18,459/81,445, 22.7%). Field completion varied systematically depending on the patient’s marital status, WHO HIV clinical stage, and family planning method in use (more effective, less effective, or no method), as well as the clinic site, study quarter, data collection form version, whether an EDD had been documented, whether an LMP had been documented, and number of weeks between the visit date and LMP. When data are missing systematically, complete case analysis can yield biased parameter estimates. We therefore chose to apply inverse probability censoring (IPC) weights to the pregnancy data before estimating incident pregnancy rates and rate ratios. In IPC, encounters with a response are reweighted by the inverse of the expected probability that the field has been completed. This is done in order to recreate the distribution for the response that would have been seen had there been no missing data. To compute the weights, we built a multiple logistic regression model for pregnancy status documentation (1 = documented, 0 = missing) as predicted by all variables identified on bivariate analysis to be associated with pregnancy documentation at p<0.20. The final model included as covariates all factors previously noted to have been associated with field completion. Encounters with a documented pregnancy status were multiplied by the model-based weights before computing aggregate counts of incident pregnancies and encounters. To account for potential variability in the computed weights given other possible sample draws from the underlying population, 95% confidence interval estimates for pregnancy rates and rate ratios were calculated by bootstrap resampling. First, 1,000 datasets of size equal to the full dataset (n = 81,445) were sampled with replacement. Next, IPC weights were estimated and regression models for pregnancy rates and rate ratios fitted to each dataset. Finally, the 95% confidence intervals for each parameter were computed from the percentiles for the parameter estimates obtained over repeated draws. To assess the sensitivity of our findings to missing data, pregnancy rates and rate ratios were also estimated from unweighted datasets. The same regression methods were used as with the weighted data, however, confidence intervals were estimated directly from the models. Only encounters with a documented pregnancy status were included in unweighted analyses.

All analyses were conducted using SAS version 9.3 (SAS Institute Inc., Cary, NC).

## Results

The demographic and clinical characteristics of HIV-infected women in participating health facilities at baseline have been previously described.[12] Briefly, women ranged in age between 18 and 45 (median = 30, IQR = 26, 36), most women were married (60%), and virtually all had completed primary school or greater (98%) (data not shown). About half (52%) of women were on antiretroviral therapy. Two-thirds (68%) were sexually active.

**Fig 2:**
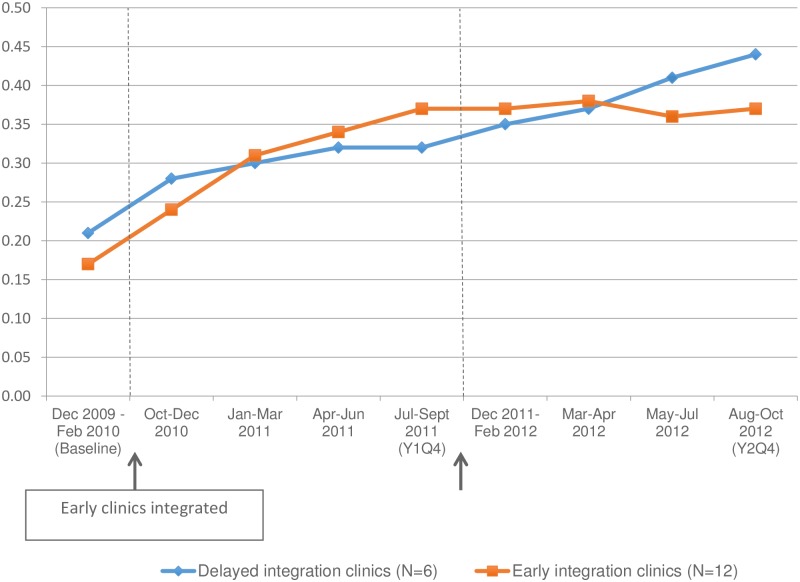
Proportion of encounters on which women used more effective contraceptive methods.

Data on the prevalence of more effective contraception among women seen at integrated and delayed integration sites are presented in Fig 2 and Table 1. Fig 2 shows more effective contraceptive use at early and delayed integration sites in two and three-month intervals over the baseline pre-integration period and two years of integration follow-up. Among the twelve early integration sites, the prevalence of more effective contraceptive methods was sustained throughout the second year of follow-up (37.5% vs. 37.0%, respectively, during Y1Q4 and Y2Q4, prevalence ratio [PR] = 0.99, 95%CI 0.90–1.08) (Table 1). Among the six delayed integration sites, use of more effective contraceptive methods rose following integration of family planning into HIV services, from 31.7% of encounters in Y1Q4 to 44.2% of encounters in Y2Q4, for an absolute increase of 12.5% (PR = 1.39, 95%CI 1.19–1.63).

**Table 1:** Prevalence of contraceptive use by method category among women’s encounters* and prevalence ratios for change in family planning method use^¥^.

Measure	Sites	Baseline	Year 1 Qtr 4	Year 2 Qtr 4	Prevalence difference between Year 2 Qtr 4 and Year 1 Qtr 4	Prevalence ratio (95%CI)	p-value
		(Dec 2009-Feb 2010)	(Jul 2011-Sep 2011)	(Aug 2012-Oct 2012)			
		%	%	%			
Using more effective family planning^†^	Early integration sites	16.7	37.5	37	-0.5	0.99 (0.90, 1.08)	0.76
	Delayed integration sites	21.2	31.7	44.2	12.5	1.39 (1.19, 1.63)	< .0001
Using less effective family planning^†^	Early integration sites	50.3	36.3	42	5.7	1.16 (1.08, 1.24)	< .0001
	Delayed integration sites	39.9	38.8	36.3	-2.5	0.94 (0.67, 1.30)	0.69
Using any family planning	Early integration sites	67	73.7	78.6	4.9	1.07 (1.03, 1.10)	0.0002
	Delayed integration sites	61.6	70.5	79.5	9	1.13 (0.94, 1.35)	0.09
Using no family planning	Early integration sites	33	26.3	21.4			
	Delayed integration sites	38.4	29.5	20.5			

*All outcomes are reported with clinic encounter as the unit of observation.

^¥^Prevalence estimates, ratios, 95% confidence intervals, and p-values are calculated from log-binomial GEE models adjusted for site-level clustering of patients.

^†^More effective family planning methods are male sterilization, IUD, subdermal implant, injectable, and oral contraceptives. Less effective family planning methods are diaphragm, condoms used without a more effective family planning method, spermicide, emergency contraception, and natural family planning methods.

In the first sensitivity analysis, we imputed IUD and implant use on every visit after first report if there were two or more reports during the observation period. As in our primary analysis, we imputed sterilization on all visits after first report. This yielded similar prevalence estimates for use of more effective contraceptives by quarter as those obtained in our primary analysis (data not shown). In our second sensitivity analysis, we analyzed contraceptive use data without method imputation. Analysis of unimputed data demonstrated a greater difference in use of more effective contraception between Y1Q4 and Y2Q4 among women seen at the delayed integration sites (absolute increase of 14.5%; PR = 1.51, 95%CI 1.21–1.88). Results for the early integrated sites were similar to those obtained from our primary analysis.

Analysis of the method mix at the early integration sites found that the prevalence of using any family planning method increased from 73.7% in Y1Q4 to 78.6% in Y2Q4 (+4.9%, p = 0.0002) (Table 1). Most of this differential increase was due to increased use of less effective contraceptive methods, which rose from 36.3% to 42.0% (+5.7%, p < .0001). Prevalence of individual more effective family planning methods remained relatively constant (Table 2). Prevalence of dual method use declined after the second year of integration, from 20.1% to 13.8% (-6.3%, p = 0.02).

**Table 2:** Prevalence of contraceptive use by method among women’s encounters* and prevalence ratios for change in family planning method use^¥^.

Measure	Sites	Baseline	Year 1 Qtr 4	Year 2 Qtr 4	Prevalence difference between Year 1 Qtr 4 and Year 2 Qtr 4	Prevalence ratio (95%CI)	p-value
		(Dec 2009-Feb 2010)	(Jul 2011-Sep 2011)	(Aug 2012-Oct 2012)			
		%	%	%			
Female or male sterilization	Early integration sites	1.6	4.1	4.7	0.6		
	Delayed integration sites	2.4	4.4	4.2	-0.2		
IUD	Early integration sites	0.3	1.1	0.7	-0.4		
	Delayed integration sites	0.1	0.6	0.5	-0.1		
Subdermal implant	Early integration sites	0.7	9.3	9.7	0.4		
	Delayed integration sites	1.8	7.6	13.1	5.5		
Injectable	Early integration sites	13	22.4	20.6	-1.8		
	Delayed integration sites	14.7	18.4	24.2	5.8		
Oral contraceptive	Early integration sites	1.2	1.8	1.9	0.1		
	Delayed integration sites	1.3	1.9	3.4	1.5		
Condom (alone or with another method)	Early integration sites	59.4	56	53.9	-2.1	0.96 (0.84, 1.11)	0.59
	Delayed integration sites	50	56.5	50.2	-6.3	0.89 (0.73, 1.07)	0.22
Dual methods (condom + injectable, oral, implant, IUD or sterilization)	Early integration sites	9.7	20.1	13.8	-6.3	0.68 (0.49, 0.95)	0.02
	Delayed integration sites	10.5	18.1	15.1	-3	0.84 (0.68, 1.04)	0.1
Other less effective method (diaphragm, spermicide, emergency contraceptive, natural methods)	Early integration sites	0.8	0.4	2	1.6		
	Delayed integration sites	0.2	0	1	1		

*All outcomes are reported with clinic encounter as the unit of observation.

^¥^Prevalence estimates, ratios, 95% confidence intervals, and p-values are calculated from log-binomial GEE models adjusted for clustering within site.

Analysis of the method mix at the six delayed integration sites found that use of any family planning method increased following integration, from 70.5% to 79.5%, although this change was not significant (+9.0%, p = 0.09) (Table 1). Use of less effective methods remained relatively constant (38.8% to 36.3%, p = 0.69). Increases in more effective contraceptive use following integration largely reflected increased use of contraceptive implants (7.6% to 13.1% in Y1Q4 and Y2Q4, respectively) and injectables (18.4% to 24.2%) (Table 2). Dual method use declined non-significantly, from 18.1% to 15.1% (p = 0.10).

Table 3 presents pregnancy rates by integration arm and year of follow-up. In total, there were 1,195 incident pregnancies among 16,689 women seen over the two years of follow-up. During the first year, the pregnancy rate was approximately 5.44 per 100 person-years (95%CI 5.12–6.62) at early integration sites and 4.87 per 100 person-years (95%CI 4.41–5.89) at delayed integration sites. During the second year, there were approximately 4.38 pregnancies per 100 person-years (95%CI 3.66–4.77) at early integration sites and 3.61 pregnancies per 100 person-years (95%CI 2.83–3.78) at delayed integration sites. The pregnancy rate ratio, comparing mean pregnancy rates for the early sites in years one and two and the delayed sites in year two with the pregnancy rate among the delayed sites prior to integration was 0.81 (95%CI 0.68–1.01). As described in the methods, this model was weighted to account for missing data. Our unweighted sensitivity analysis gave similar results (data not shown). When we restricted the analysis to examine effects of integration under Kenya Ministry of Health management, averaging pregnancy rates across the early and delayed integration sites during the second year of observation compared to the pregnancy rate among the six delayed integration sites in year one, the weighted pregnancy rate ratio was 0.72 (95%CI 0.60–0.87). The unweighted analysis yielded a pregnancy rate ratio of 0.72 (95%CI 0.52–0.99).

**Table 3:** Pregnancy rates and rate ratios during years one and two of integration follow-up.

Year	Sites	n pregnancies	n women (n clinic visits*)	estimated person-years of follow-up**	IPC-weighted pregnancy rate per 100 person-years of follow-up^҂^ (95% CI)	IPC-weighted pregnancy rate ratio҂ (95% CI)
Year 1	Early integration sites	324	4446 (15489)	5235	5.44 (5.12, 6.62)	
	Delayed integration sites	352	3664 (14680)	4961	4.87 (4.41, 5.89)	
Year 2	Early integration sites	279	4500 (16173)	5466	4.38 (3.66, 4.77)	
	Delayed integration sites	240	4079 (16644)	5626	3.61 (2.83, 3.78)	
Early integrated sites year 1 and all sites year 2 vs. delayed integrated sites year 1						0.81 (0.68, 1.01)
All sites year 2 vs. delayed integrated sites year 1						0.72 (0.60, 0.87)

*Figures exclude 18,459 visits missing pregnancy status.

**n clinic visits has been multiplied by the mean interval between clinic visits (0.338 years) during the follow-up period among women seen at sites where all visits were entered into OpenMRS.

^҂^Pregnancy rates and rate ratios were calculated from negative binomial GEE models adjusted for clustering within site. Inverse probability censoring (IPC) weights have been applied to correct estimates for potential biases related to differential documentation of pregnancy status.

## Discussion

Integration of family planning services into HIV care clinics increased use of more effective contraceptive methods,[12] which appears to persist up to 24 months following implementation of the integrated service model. We note that the delayed intervention group experienced an increase use of more effective contraception per visit during year 1 of follow-up which likely reflects the systems strengthening performed by the study team at all 18 health facilities including: training of health staff, support for commodity procurement, supervision of provision of family planning, etc. Thus, the difference in contraceptive use between the two study arms, and sustained in the second year of follow-up reflects the different service delivery models, intervention vs. control.

During the second year of observation, the Ministry of Health successfully implemented the integrated service model with minimal external supervision from FACES technical advisors, demonstrating sustainability and reproducibility of the intervention. Our prior analysis of the cost, cost-effectiveness and cost-efficiency of integrated family planning and HIV services was limited to the first 12 months of observation.[19] Although we did not collect cost data during the second year of observation, training and supervisory needs decreased substantially while the number of prevented pregnancies increased. Thus, we suspect that integration of family planning and HIV services likely leads to even greater cost-effectiveness than previously reported.[19]

The “one-stop-shop” model of family planning and HIV service integration led to a 19% to 28% decrease in pregnancy incidence over the 24-month follow-up period (Table 3). To our knowledge, this represents the first evidence that integration leads to decreased pregnancy among HIV-infected women. We note that the decline in pregnancy was only significant when the pregnancy rate at early integration sites in year 1 were not included in the model. Furthermore, due to the nature of the study conducted utilizing Ministry of Health medical records, we were unable to collect data on pregnancy intention. We know that the unmet need for contraception in the Nyanza region is high (23.2%).[20] Still, during this two-year period of follow-up, we are not aware of policies or other factors that would have significantly affected pregnancy intention, availability and use of family planning services in the study region. Globally, contraceptive use prevents more than 200 million unintended births annually, which lowers rates of both unintended pregnancy and abortion and may significantly lower maternal and infant mortality rates as well.[20] Thus, the integration of family planning services may ultimately help HIV-affected couples to achieve their desired family size. Future research should examine the potential spillover effect of family planning and HIV service integration to improve contraceptive uptake and continuation among HIV-uninfected women. For example, the systems strengthening, staff training and improved commodity management required to successfully integrate services may have an overall impact on family planning provision for an entire facility or health system and ultimately lead to lower national fertility rates.

Dual method use initially increased from baseline to the end of year 1 at both early and delayed integration facilities. However, of some concern, dual method use declined significantly at early- and non-significantly at the delayed integration clinics by the end of year 2. In a recent study conducted in three African countries including Kenya, dual method use was reported by 31% of HIV-infected women, but was lower among women using more effective contraceptive methods.[21] In another recent survey, 48% of women in Kenya surveyed were found to have an unmet need for dual method use based on their desire to prevent pregnancy and absence of condom use at last sex.[22] While HIV testing continues to expand in Kenya, a survey from 2012 in Kenya found that only 32% and 44% of reproductive aged men and women, respectively, in the Nyanza region reported ever being tested for HIV. Thus, many HIV-infected persons in Kenya are unaware of their HIV status, posing a major barrier to HIV prevention[23] and pointing to the need for continued condom use to prevent HIV transmission. In certain circumstances, for example in known concordant HIV-positive couples and in cases of HIV viral suppression among the HIV-infected person in a mutually monogamous discordant couple [24] condom use may be redundant for women using more effective contraceptive methods. Nevertheless, the trend of decreasing condom use among women using more effective contraceptive methods is concerning, and demonstrates the need for further research in this area. For example, development of counseling strategies that incorporate condom promotion among women choosing more effective contraceptive methods may help to decrease the transmission of HIV and sexually transmitted infections while preventing unplanned pregnancy.

In addition to the original cluster-randomized design, strengths of this study include the system-strengthening training that was performed at both early and delayed integration sites to isolate the effectiveness of integration. Most importantly, the Kenyan Ministry of Health led the integration at the six delayed sites, and maintained the integrated model at the 12 early integrated sites, with limited support from the FACES’ technical assistance team. We also included facilities of different levels of care including district hospitals, health centers and dispensaries. The study has several limitations. Condom use was based on self-report, and social desirability bias may have increased reported consistent use. However, this bias is likely to be similar in both study arms. In addition, a small fee ($0.54 –$1.09) intermittently charged to patients seeking contraception at three of the delayed integration facilities might have reduced use of more effective methods at these sites. There are additional limitations related to our pregnancy data, including the fact that not all incident pregnancies (especially early pregnancies) were likely detected during the follow-up period, and we did not have information on pregnancy intendedness. Unfortunately, our study period overlapped with a transition to a new patient encounter form, the Ministry of Health 257 ‘Blue Card’, which no longer collects information on patient sexual activity. Thus, while controlling for differences in sexual activity by time point would indeed be sensible, we did not have the data to do so. Finally, our findings are specific to the model of family planning—HIV integration studied.

The “one-stop-shop” approach to integration of family planning services into HIV care clinics led to a durable and sustained increase in the use of more effective contraceptive methods, and to a decrease in pregnancy incidence 24 months following implementation of the integrated service model. Additionally, our data show that integration led and maintained by the Kenya Ministry of Health yielded results similar to those observed in the initial CRT, highlighting reproducibility of the intervention and feasibility of health ministry led scale-up of the “one-stop-shop” integrated service model outside of a research-driven implementation effort. In addition, our team collaborated with the Ministry of Health to help develop and disseminate a Sexual & Reproductive Health/HIV Service Integration Toolkit for use throughout the country. In many settings in sub-Saharan Africa, while women have the right to make contraceptive choices to control their fertility, structural barriers often lead to unintended pregnancy. We believe that integration of services decreases the barriers to contraception among HIV-infected women, and creates an environment at the clinic that encourages women to use their agency to make contraceptive choices based on their reproductive desires and health needs.

## Supporting information

S1 File Study protocol. (PDF)

S2 File Consort checklist cluster RCT. (DOCX)
