# Supplementary material for: Integration of family planning services into HIV care clinics: Results one year after a cluster randomized controlled trial in Kenya
Source: PLoS One. 2017 Mar 22;12(3):e0172992. doi: 10.1371/journal.pone.0172992 (PMC5362197; doi:10.1371/journal.pone.0172992)
Supplement: S1 File — (PDF) [file pone.0172992.s001.pdf]

Cluster randomized trial of integration of Family Planning Services into HIV Care and Treatment in Suba, Kisumu East, Rongo and Migori districts of Nyanza Province

Name	Organization	Role on Project
Craig Cohen	University of California, San Francisco, Department of Obstetrics, Gynecology, and Reproductive Sciences, Bixby Center for Global Reproductive Health	Principal Investigator
Elizabeth Bukusi	Center for Microbiology Research, Kenya Medical Research Institute	Co-Principal Investigator
Daniel Grossman	Ibis Reproductive Health	Co-Principal Investigator
Sara Newmann	University of California, San Francisco, Department of Obstetrics, Gynecology, and Reproductive Sciences	Co-Investigator
Starley Shade	University of California, San Francisco, Department of Medicine, Center for AIDS Prevention Studies	Co-Investigator
Katie Doolan	University of California, San Francisco, Department of Obstetrics, Gynecology, and Reproductive Sciences, Bixby Center for Reproductive Health	Co-Investigator
Maricianah Onono	Center for Microbiology Research, Kenya Medical Research Institute	Co-Investigator
Betty Njoroge	Center for Microbiology Research, Kenya Medical Research Institute	Co-Investigator

COLLABORATING INSTITUTIONS

University of California San Francisco: UCSF is the only University of California campus dedicated exclusively to the health sciences and is home to graduate professionals in medicine, nursing, pharmacy, and dentistry, and a graduate division for pre-doctoral and postdoctoral scientists. In addition to its main campus, where the Schools of Medicine, Nursing, Pharmacy, and Dentistry and the UCSF Medical Center are located, UCSF encompasses several major sites in San Francisco, including UCSF Mount Zion, the Comprehensive Cancer Center, and Mission Bay. UCSF is considered one of the nation’s premier health sciences, training, and research centers and has a well-established reputation in biomedical research.

Kenya Medical Research Institute (KEMRI): The Kenya Medical Research Institute (KEMRI) is one of the leading health research institutes in Africa. KEMRI was established in 1979 under the Science and Technology (Amendment) Act of that year to represent the national body responsible for carrying out health science research in Kenya. KEMRI’s mission is to conduct health research and generate research findings to be applied towards improvement of health in Kenya and the world over. Under the Amendment Act, the KEMRI was charged with the responsibility of carrying out health research with the following mandates:

- To carry out research in the field of biomedical sciences;
- To co-operate with other organizations and other institutions of higher learning in training programs and on matters of relevant research;
- To liaise with other research bodies within and outside Kenya carrying out similar research;
- To disseminate research findings;
- To co-operate with the Ministry of Health, the Ministry responsible for research, the National Council for Science and Technology, and the Medical Science Advisory Committee on matters pertaining to research policies and priorities.

With health research and training expertise in infectious disease, parasitic disease, epidemiology, and biotechnology and non-communicable diseases, KEMRI has grown to become one of the leading centers of excellence in health research development and Africa's largest health research institute. KEMRI has trained cadres of professionals and maintains over 80 professors with PhD degrees or equivalent, over 148 with MA or equivalent, and nearly 400 highly trained and skilled technical staff. As a partner in global health initiatives, KEMRI collaborates with the World Health Organization (WHO), the Japan International Cooperation Agency (JICA), U.S. Center for Disease Control and Prevention (CDC), among others. KEMRI serves as the Collaborating Center for HIV/AIDS, Tropical Research, Polio Immunization, Viral Hemorrhagic Fevers, and Anti-Microbial Resistance.

Research, Training, and Care Program (RTCP), KEMRI: Drs. Bukusi and Cohen with others established the Research, Training, and Care Program (RTCP) with KEMRI and the University of Nairobi in 1994. RTCP was established to facilitate collaborative research on STIs, as well as HIV prevention, care, and treatment between investigators from the US and Kenyan counterparts in reproductive tract infections at both Kenyan institutions. The RTCP currently involves the Center for Microbiology Research and the Center for Respiratory Diseases Research at KEMRI; and at the University of Nairobi, departments include Obstetrics and Gynecology, Community Medicine, and Medical Microbiology. The RTCP provides fiscal and administrative support and office space to numerous research studies and programs funded by the NIH, CDC, WHO, and PEPFAR/CDC. The RTCP expanded in recent years to include the KEMRI-UCSF Infectious Disease Research Training Program (IDRTP) which is training three PhD and five Masters students from Kenya in STI research over the next five years. In addition to the IDRTP students, RTCP has trained a UCSF Reproductive Infectious Disease fellow, several Kenyan physicians leading to the MPH degree at the University of Washington, and a large numbers of U.S. medical students, residents and other research staff including biostatisticians, behavioral scientists and laboratory scientists. A number of other Kenyan physicians training in the Department of Obstetrics and Gynecology at the University of Nairobi have also received support in conducting their research projects to complete the requirements for the Masters of Medicine.

UCSF Department of Obstetrics/Gynecology and Reproductive Sciences: The Department boasts a diverse portfolio of research activities, from basic biological, translational, and clinical investigation to epidemiological inquiry and the development of public health policy. The Department is home to the UCSF National Center of Excellence in Women's Health. The Department's exemplary academic reputation (consistently among the top three departments of obstetrics and gynecology in the nation to receive federal funding) derives from long-standing integrative and multidisciplinary partnerships within the UCSF community of nursing and medical scholars.

Bixby Center for Global Reproductive Health, UCSF: The UCSF Bixby Center for Global Reproductive Health was formed in 1999 to address the health, social, and economic consequences of sex and reproduction through research and training in contraception, family planning, and STIs. The Bixby Center strives to develop preventive solutions to the most pressing domestic and international reproductive health problems. The Bixby Center is currently carrying out over 100 domestic and international projects in the areas of abortion and post-abortion care, adolescent sexuality and reproductive health, contraceptive development and family planning, HIV/AIDS and other STIs, safe motherhood, and unintended pregnancy.

Ibis Reproductive Health: Ibis Reproductive Health is a non-governmental organization that aims to improve women's reproductive autonomy, choices, and health worldwide by conducting original clinical and social science research, leveraging existing research, producing educational resources, and promoting policies and practices that support sexual and reproductive rights and health. The organization was founded in 2002 to support talented new clinical and social science researchers committed to studying critical, yet often overlooked, topics in the reproductive health field. With the promotion of women's sexual and reproductive autonomy as the guiding principle of its work, Ibis's staff tackles the full spectrum of cutting-edge reproductive health and rights topics in the U.S., Sub-Saharan Africa, Latin America, and the Middle East and North Africa. Ibis's staff of roughly twenty is located in offices in Cambridge, Massachusetts (headquarters); Johannesburg, South Africa; and San Francisco, California.

ABSTRACT

Approximately three quarters of the world's population living with HIV/AIDS live in Sub-Saharan Africa, and 60 percent are estimated to be women. Globally, fourteen million children have lost one or both parents to HIV/AIDS, and it is estimated that 80% of these children orphaned from HIV/AIDS live in Sub-Saharan Africa[1]. Additionally, more than 90% of all pediatric HIV infections are due to maternal-child transmission of HIV, and more than 90% of these pediatric infections are in Sub-Saharan Africa[2]. Increased use of family planning among HIV-infected individuals would help to decrease the number of unintended pregnancies among HIV-infected women thereby decreasing both the number of children who are orphaned from HIV/AIDS and the number of children who are infected with HIV. Additionally, decreasing unintended pregnancies among HIV-infected women in Sub-Saharan Africa is expected to decrease maternal mortality. Maternal mortality in several Sub-Saharan African countries has increased as HIV prevalence among reproductive age women has risen[3].

In 2003 the President's Emergency Plan for AIDS Relief (PEPFAR) initiative was created to increase HIV prevention and treatment programs in 15 countries in Africa. Since 2004 there has been a rapid scale-up in treatment programs in all PEPFAR-funded African nations. Unfortunately family planning services has not been included in this initiative, leaving HIV-infected women to seek family planning services elsewhere. Several national and international conferences have been held focusing on the integration of family planning services into HIV care and treatment. The consensus among multiple local and international public health organizations and funding agencies is that integration of family planning and HIV care in Sub-Saharan African countries is of utmost importance in order to decrease the rate of HIV infection transmission and the societal impact of HIV infection[4-9]. Despite the recent international attention to the idea of integrating family planning into HIV care and treatment, few studies have evaluated the most effective models of integration and their outcomes. We propose conducting a cluster randomized trial comparing the integration of family planning services into HIV care and treatment programs versus the standard referral for family planning services outside of HIV care and treatment programs within Suba, Kisumu East, Rongo and Migori districts in Nyanza province;.

The objectives of this research are:

1. To assess the impact of integrating family planning services into HIV care and treatment on the utilization of family planning by HIV-infected women and men.
2. To estimate the impact of integrating family planning services into HIV care and treatment on unintended pregnancies among HIV-infected woman; and
3. To assess the feasibility and acceptability of integration of family planning into HIV care and treatment programs.

We hypothesize that the integration of family planning services into HIV care and treatment will increase the utilization of more effective contraception (MEC) (including oral, implantable, injectable, and intra-uterine methods) among HIV-infected women. We hypothesize that by increasing the utilization of MEC, contraceptive compliance and continuation among HIV-infected women will increase and unintended pregnancy rates among HIV-infected women will decrease. This study will help us understand the acceptability and feasibility of integrating family planning services into HIV care and treatment programs. It will also help describe the effects of integration on contraceptive use, unintended pregnancies, and HIV care.

BACKGROUND AND JUSTIFICATION

There is a need for increased family planning services for people living with HIV infection in Sub-Saharan Africa.

Recent studies suggest a large unmet need for contraception among HIV-infected women in Africa receiving antiretroviral therapy (ARV)[9]. Family planning services, however, remain separate from HIV care and treatment services, leaving many HIV-infected women without resources for preventing pregnancy. The integration of family planning services into HIV care and treatment programs would have multiple public health benefits by preventing adverse consequences of pregnancy for HIV-infected women and by providing women with the opportunity to make reproductive choices.

The paradigm of integrating family planning into HIV services is being promoted by multiple international public health agencies and foundations, as well as local organizations. This public health initiative could result in fewer unintended pregnancies, and therefore decrease mother-to-child HIV transmission, illegal abortion, maternal and neonatal morbidity and mortality, and various other health and societal cost outcomes related to unintended pregnancies and vertical transmission of HIV[4, 5]. Despite this new interest in the integration of family planning into HIV services, very few studies have evaluated the clinical impacts of integration. By decreasing the pregnancy rate in HIV-infected women, family planning services are expected to decrease maternal mortality rates in Sub-Saharan Africa, which have risen alongside increases in HIV prevalence[3]. Similarly, by decreasing the pregnancy rate in HIV-infected women, family planning services would help to decrease neonatal morbidity and mortality, such as stillbirth, low birth weight, and prematurity, all of which are common among infants of HIV-infected women[10].

As in much of Sub-Saharan Africa, actual fertility among women in Kenya is significantly higher than wanted fertility[11]. In Kenya and Zambia, over 50 percent of births over a five and three-year period, respectively, were found to be unintended, many to women with unknown HIV status[12, 13]. In Uganda 97 percent of pregnant, HIV-infected women who had been offered antiretroviral treatment (ART) in rural Uganda did not want to be pregnant[9]. Despite the high rates of unintended pregnancy, contraceptive prevalence in sub-Saharan Africa is low, with only 13% of married, reproductive-aged women using contraception in 2002[14].

People living with HIV/AIDS, policy makers, and service providers feel that integration of family planning into HIV care is the logical solution to meet the unmet need for family planning services. In Uganda, a group of people living with HIV/AIDS desired increased access to contraception and preferred to receive family planning services where they receive HIV care. They reported preferring providers who already knew them and not having time to access other services at a different location[15]. Women reported feeling that integrated services would help them change their partners' negative views on family planning since they could attend sessions together[16]. In Kenya, the majority of HIV care providers interviewed in a study reported a high unmet need for family planning among HIV-infected individuals because of reluctance to visit family planning clinics due to negative attitudes among providers toward people with HIV[6]. Policy makers and providers in Kenya view the rational next step to be integration of family planning into HIV services, believing that the integration of the two could complement each other, reduce cost and time for clients and services, and improve client follow-up[6]. Taken together, these studies suggest that HIV-infected patients, HIV-care providers and local policy makers all see a need to expand family planning services for HIV-infected individuals in Sub-Saharan Africa.

This proposed study will be conducted in the Suba, Kisumu East, Rongo and Migori districts of the Nyanza Province of Kenya. It will be conducted within the Family AIDS Care and Education Services (FACES), which is funded primarily through the US President's Emergency

Plan for AIDS Relief (PEPFAR) via the Center for Disease Control (CDC). FACES began services in March 2005 in Nyanza Province and is a collaboration between the Kenya Medical Research Institute (KEMRI) and the University of California, San Francisco (UCSF). FACES' mission is to provide accessible comprehensive care and support services to HIV affected families and other vulnerable populations, reinforce prevention practices, and build capacity related to HIV/AIDS care. FACES partners with government health facilities to provide HIV care, treatment, and support to individuals and families in the Nyanza province and currently has over 13,000 patients enrolled in care and over 3,000 on antiretroviral therapy (ART). The Nyanza province has the highest HIV prevalence in Kenya, with 18.3% of women and 11.6% of men aged 15-49 estimated to be infected in 2003[15]. FACES data suggests that contraception prevalence ranges somewhere between 23-35 percent[17]. Because family planning services are not currently provided in an integrated manner within the care facilities that FACES currently works in or those where it will expand to within the 2007-2009 fiscal years in Suba, Kisumu East, Rongo and Migori Districts, this setting affords an excellent opportunity to study the effect of integration on critical indicators of contraceptive use.

There are data to support the hypothesis that integration of family planning into HIV care would be effective.

Several studies have shown that integrating family planning services into HIV voluntary counseling and testing increases contraceptive use and decreases unwanted pregnancies[18-20]. Additionally, a few theoretical and/or cost-effectiveness studies have estimated that provision of family planning to HIV-infected women via integration of family planning into HIV care programs would decrease unintended pregnancies and maternal-child HIV transmission rates, and would be cost-effective. One study demonstrated that moderate reductions in unintended pregnancies rates of HIV-infected women (estimates varied by country) would result in the equivalent number of HIV-infected births averted as would current prevent mother-to-child transmission (PMTCT) efforts using Nevirapine [21]. Another model demonstrated that integration of family planning into PMTCT services could avert approximately two times the number of child HIV infections (71,000 versus 39,000) that current PMTCT services are estimated to prevent[22]. A cost-effectiveness analysis concluded that providing family planning and increasing contraception use among HIV-infected women who do not want to be pregnant is as cost-effective as current PMTCT programs providing Nevirapine and averts almost 30 percent more HIV-positive births than does PMTCT programs with Nevirapine [23]. Thus, there is experience with combining family planning with voluntary counseling and testing services, and there are projected data to show that integration of family planning and HIV care services would succeed at decreasing both unintended pregnancies and HIV transmission, and would be cost-effective.

Preliminary data

In 2006, we performed an internal service provision improvement evaluation of the reproductive health services at FACES clinics[24]. The evaluation consisted of 24 structured observations of all types of client interactions, including Voluntary Counseling and Testing (VCT), HIV (peer) education, adherence counseling, enrollment visits, clinical and non-clinical follow-up visits, and family counseling sessions.

Several barriers to effective reproductive healthcare were identified: environmental barriers (related to space, examining equipment, contraceptive methods available, cultural discomfort, technical discomfort with gynecologic exams); lack of inclusion of reproductive health history into enrollment and follow-up visits; lack of inclusion of family planning counseling/care into routine clinic visits; long intervals between client request for family planning and actual method initiation; and lack of family planning counseling training/experience among clinic staff.

The evaluation revealed that only 20% of the observed clinical encounters involved any family planning (FP) counseling and only 16% of clinical encounters involved spontaneous, provider-initiated FP information. The content of the FP counseling varied greatly. Despite a space on the intake form for the date of the last menstrual period, frequently when answers of greater than one month were given, an evaluation for pregnancy was not performed. This inadequate response to delayed menses raises the concern of the occurrence of unintended pregnancies, potential teratogenicity of HIV medication when pregnancy is not detected, and missed opportunities for early referral to antenatal care. Additionally, despite prompts on the clinical intake forms, providers were observed to inquire about sexual activity and contraception use only approximately 30% of the time. Only three of the observed clinical encounters resulted in steps towards contraception initiation and only one of the 24 observed clinical encounters involved any discussion of screening for risks involved with different contraceptive methods.

This assessment of reproductive health services at FACES is limited by a small sample size and non-standardized method of observation and evaluation. However, certain themes do emerge having to do with inadequate counseling and assessment of patient's reproductive needs/desires, insufficient technical support with offering reproductive health services, inadequate training in family planning counseling for clinicians, and scarce method supply and ability to initiate contraception at time of discussion of patient's need/desire to contracept. These findings blatantly illustrate the need for improvement of family planning and other reproductive health services within the FACES HIV care and treatment programs. It is also likely that these qualitative findings from the FACES centers would be similar at other HIV care and treatment programs throughout Kenya and Sub-Saharan Africa.

HYPOTHESIS

We hypothesize that the integration of family planning services into HIV care and treatment will increase the utilization of all modern contraceptive methods, especially more effective contraception (MEC) (including oral, implantable, injectable, and intra-uterine methods), among HIV-infected women. Additionally, we hypothesize that by increasing the contraceptive prevalence, contraceptive compliance and continuation among HIV-infected women will increase and unintended pregnancy rates among HIV-infected women will decrease.

GENERAL OBJECTIVES

The general objective of this study is to determine whether an integrated model of family planning provision at HIV care and treatment centers better addresses the family planning needs of HIV-infected people and their partners than the existing non-integrated model. The results of the study will provide information for policy makers and program planners that will help them implement high quality family planning services for this population.

SPECIFIC OBJECTIVES

1. To compare the contraceptive prevalence among HIV-infected individuals receiving care at PSCs randomized to the integrated model to that of HIV-infected individuals at control (non-integrated) PSCs
2. To compare the proportion of pregnancies that are unintended among HIV-infected women receiving care at PSCs randomized to the integrated model to that of HIV-infected women at control (non-integrated) PSCs

3. To assess the feasibility and acceptability of integration of family planning into HIV care and treatment programs;
4. To evaluate knowledge of contraception and the importance of dual method use among HIV-infected women;
5. To better understand the pregnancy intentions of HIV-infected women and men who seek health care services in Migori, Rongo, Kisumu East and Suba Districts

STUDY DESIGN/METHODOLOGY

Overview

The study will have three parts. During the first part (3 months), baseline data on contraceptive prevalence and unintended pregnancy will be collected at 18 PSCs that are supported by the FACES program in Suba, Kisumu East, Rongo and Migori Districts. Also during this first part, information about knowledge, attitudes and practices related to family planning will be obtained from patients and from clinicians at the sites. During the second part (3 months), an intervention consisting of integrating family planning services will be designed and implemented at twelve of the 18 FACES-supported PSCs. We aim to utilize a 2:1 integration:control model, with delayed integration so as to gradually expand the integration of FP and HIV services throughout the 18 sites, while maintaining the ability to test hypotheses on the effects of integration on health outcomes. After collecting the baseline data, we plan to stratify the clinics into two categories based on the overall patient volume and differences in the structure of family planning provision, i.e., whether or not there is a specific MCH division providing family planning at the site or not. Randomization of sites will then occur among clinics in each of these strata, with a ratio of two intervention sites (integrated model) to one control site (family planning provided in the standard manner at the site). During the third and last part (9 months), data on contraceptive prevalence and unintended pregnancy will be collected from each of the 18 sites. At the end of part three, information about knowledge, attitudes and practices related to family planning again will be obtained from female patients and from clinicians at the sites. In addition, we will administer questionnaires assessing the acceptability of family planning services to patients, and in addition clinic staff will be answer a questionnaire assessing the feasibility of providing integrated family planning services.

Study population/Study subjects

The study population will primarily consist of HIV-positive men and non-pregnant, HIV-positive women of reproductive age who obtain care at the FACES-supported PSCs in Suba, Kisumu East, Rongo and Migori Districts in Nyanza Province, Kenya. In addition, study subjects will also include clinic staff at the FACES centers included in the study.

Inclusion / exclusion criteria for sites

Eighteen PSCs in Suba, Kisumu East, Rongo and Migori Districts will be selected from existing public heath facilities. The existing facilities include sub-district referral hospitals, health centers, and dispensaries. Inclusion in the study will be based on the following criterion: each site must be providing HIV care and treatment services to non-pregnant women of reproductive age. Sites will be excluded if they do not meet the inclusion criterion listed above or if they are already providing integrated comprehensive HIV care and treatment that includes on-site family planning counseling and provision.

1. Part I: Baseline data collection (Months 1-3)

1.1. Baseline site-based data collection

During a period of three months, baseline quantitative data on contraceptive prevalence, contraceptive methods used and pregnancy (including degree to which pregnancy was planned and desired) will be collected as part of routine clinical care at the 18 sites in Suba, Kisumu East, Rongo and Migori Districts. This information will be collected in a standardized clinical encounter form from all HIV-positive women age 18-45 at the time of each visit to the site. Data will also be collected on HIV-positive men age 18-45 regarding condom use, vasectomy and partner's use of other methods. A draft of the questions that will be used to collect the site-based data is included in Appendix A.

1.2. Baseline knowledge, attitudes and practices (KAP) survey of female and male clients

Also during this three-month period, a convenience sample of approximately 28 female patients and 28 male patients will be recruited from each of the 18 sites (N=488 female and 488 male patients) to complete a questionnaire regarding knowledge, attitudes and practices (KAP) related to family planning. The purpose of the KAP survey is both to 1) measure the satisfaction with the family planning services offered at the HIV care and treatment clinic, 2) provide information that will be used to design the family planning integration intervention and improve family planning provision, and 3) provide a baseline measure of KAP that will be compared to follow-up surveys at the end of the study.

The survey will be administered face-to-face by an interviewer in the respondent's preferred language in a private area of the centre.

The inclusion criteria for the client KAP survey will be:

1) HIV-positive woman or man obtaining care at the FACES site
2) Not currently pregnant (if female)
3) Age 18 to 45 years old if female; age 18+ if male
4) Willing and able to give informed consent

Clients will be excluded if they do not meet the inclusion criteria listed above. Some of the topics included in the KAP survey will be: familiarity with contraceptive methods; prior and current contraceptive use; partner's role in contraceptive decision-making; interest in preventing pregnancy in the future; and acceptability of and satisfaction with currently-offered family planning services at the site. The survey will be adapted from the Demographic and Health Survey modules on contraception, marriage and sexuality, and fertility preferences (see Appendix B1-1 for women's survey and Appendix B1-2 for men's survey) We will train our interviewers to provide referrals for women who need counseling or support services to a local women's non-governmental organization (NGO) working in Migori District, the Women's Action Forum for Networking (WAFNET). These referrals will be provided to women who report domestic abuse (psychological, physical, or sexual) or other negative events during the interviews. .Participants will be reimbursed a maximum of USD$3 (approximately 200 Kenyan Shillings at current exchange rate) for transport.

1.3. Baseline open-ended interviews with female and male clients

During the three-month baseline period, we will perform 30 open-ended interviews with female patients recruited from the 18 PSC sites. We will also perform 30 open-ended interviews with male patients recruited from the same sites. These patients will be a subset of those participating in the KAP questionnaire. The purpose of these interviews will be to obtain qualitative information about their reproductive intentions and their

perceived barriers to obtaining and using effective contraception, information that cannot be easily obtained using the survey methodology. These data will be used to design the family planning integration intervention. The final version of the interview guides will be submitted to the IRB prior to initiating data collection.

A convenience sample of 30 women and 30 men will be invited to participate in the open-ended interview. The open-ended interview will be performed in the respondent's preferred language in a private area of the centre and will take approximately 30 minutes to complete. The inclusion criteria will be:

1) HIV-positive woman or man obtaining care at the PSC site
2) Not currently pregnant (if woman)
3) Age 18 to 45 years old if female, age 18+ if male
4) Has not received vasectomy or bitubal ligation
5) Willing and able to give informed consent

Subjects will be reimbursed a maximum of USD$3 (approximately 200 Kenyan Shillings at current exchange rate) for transport.

All interviews will be tape recorded (if the woman gives informed consent), transcribed, translated to English, and a content analysis will be performed using the qualitative software ATLAS.ti.

1.4. Baseline survey with clinic staff at PSC sites

During the three-month baseline period, we will also administer a survey to approximately 30-45 clinic staff at HIV care and treatment sites, including doctors, nurses, clinical officers and peer counselors (convenience sample of approximately 3-4 at each site). The purpose of the KAP survey is both to 1) provide information that will be used to design the family planning integration intervention in the subsequent study, and 2) provide a baseline measure of KAP that will be compared to follow-up surveys of the same clinic providers at the end of the study. The final version of the survey will be submitted to the IRB prior to initiating data collection.

The survey will be administered by an interviewer in the respondent's preferred language in a private area of the centre. Inclusion criteria for the clinic staff survey will be: 1) clinic staff at the FACES-supported PSC site with direct patient contact, and 2) willing and able to give informed consent. Clinic staff will be excluded if they do not meet the inclusion criteria listed above. Some of the topics included in the survey will be knowledge of contraceptive methods and appropriate counseling, prior experience providing methods and interest in playing a more active role in contraception provision. Open-ended questions will attempt to obtain qualitative information about the perceived barriers to providing contraception in the sites. The responses to these open-ended questions that will be tape recorded (if the participant gives his/her consent), transcribed, translated to English (if interview not performed in English), and a content analysis will be performed using the qualitative software ATLAS.ti. Clinic staff will be reimbursed with a book voucher worth approximately USD$5 (approximately 350 Kenyan Shillings at current exchange rate) for participating in the survey.

2. Part II: Intervention design and implementation (Months 4-6)

The study will be conducted as a cluster-randomized trial. Eighteen clinical sites in the Suba, Kisumu East, Rongo and Migori Districts will be randomized to a family planning intervention or to control. Sites will be grouped into strata based on the overall patient

volume and differences in the structure of family planning provision. The randomization scheme will be generated using STATA statistical software. Below we briefly outline the design and implementation of the intervention.

2.1. Intervention design

The information gathered during the baseline data collection period will serve to inform the ultimate design of the family planning intervention. During the Hormonal Contraception and HIV: Science and Policy Africa Regional Meeting in Nairobi in 2005, one of the important areas for further research identified was a "better understanding of how effectively to promote dual protection, especially optimal counseling processes and techniques.[25]" Additionally, a needs assessment performed in Uganda[26] prior to integrating family planning into HIV services there identified several issues, among them were: the need to include men in family planning education/counseling, fear of side effects of contraception among rural HIV-infected individuals; lack of family planning training among health care providers; and concern among some health care provides that promoting contraception will encourage HIV infected clients to have sex. Our intervention will take into account the areas identified in the Nairobi meeting and in the Uganda needs assessment in addition to the Nyanza-specific needs assessment that will be conducted during Part 1 of the project. In planning our intervention, we will aim to create an optimal way to integrate family planning counseling, family planning method provision and dual method promotion into HIV care and treatment services.

The intervention to be designed will include four major components of care: patient education; family planning counseling; method provision, including risk assessment; and contraception follow-up. The details of the intervention will greatly depend on the information gathered during the qualitative interviews with clients and clinicians, as well as the results of the KAP surveys. With these results, the research team will be able to tailor the design of the intervention to meet the needs of patients and providers in the Nyanza Province. Particular attention will be paid to results regarding: myths about and barriers to family planning; methods preferred by patients; feasibility of providing long-acting methods such as implants and intrauterine contraception; preference for types of locations and clinicians from whom patients feel most comfortable receiving family planning; and clinicians' concerns regarding assessments of family planning needs and provision of methods. With regards to method provision, the intervention will incorporate the Kenyan *Family Planning Guidelines For Service Providers* written and revised by the Kenyan Ministry of Health's Division of Reproductive Health in 2005[27]. The integration model will aim to address needs identified during the baseline data collection period and, as appropriate, will be loosely modeled after existing family planning and HIV care integration programs in Uganda, Zambia, and Zimbabwe [28].

Specifically, the intervention will consist of:

- Patient Education:
  - Group and individual education sessions geared toward:
    - Explaining importance of dual method protection: differentiating the importance of condom use for prevention of STI transmission from the importance of family planning for pregnancy prevention
    - Explaining the different methods available (pills, injections, implants, intrauterine devices, sterilization), how they work, and potential side effects associated

        - Tailored client education materials focusing on available methods, importance of contraception and dual method use, and side effects of different methods will be created in the forms of low literacy pamphlets, flip charts, and posters.

- Family planning counseling:
    - Extensive provider and health counselor training in family planning including:
        - How to incorporate family planning counseling into HIV enrollment and follow-up visit (inquiring at each visit as to patient's contraceptive needs, questions, etc.)
        - The importance of contraception for pregnancy prevention
        - Side effects of contraception
        - Risks of contraception
        - The importance of dual method use to prevent transmission of STI's
        - The significance of preventing unintended pregnancies with regards to prevention of maternal-to-child HIV transmission
        - Training on individual and group family planning counseling techniques, including an assessment of patients' contraceptive needs/desires in the context of their lives and health situations
        - Education on contraception options, contraceptive risks and side effects, and prescription and dispensing of contraception
        - Promotion of male partner involvement in family planning education and choices

- Method Provision:
    - Contraception will be prescribed directly by HIV care and treatment program clinicians. Commodities will be obtained through the normal Ministry of Health procurement process.

    - Education for providers regarding individual patient risk assessment based on client's health status and current co-morbidities
        - Examples:
            - Risk of thrombosis for patients who have a history of thrombosis, who smoke, etc.
            - Contraceptive interactions with ART medications, antibiotics, anti-tuberculosis medications, anti-seizure medications, etc.

    - Education for providers regarding dosing of hormonal contraception, timing of initiation (including education about and promotion of "Quick Start" methodology), and interval follow-up and repeat dosing (for injectables)

    - Training on intrauterine contraception and implant insertion at clinics where provision of these methods is feasible.

- Contraception Follow-up
    - Training of providers regarding method-specific follow-up:
        - appropriate timing of follow-up
        - questions about side effects, method satisfaction
        - follow-up physical exam (if method appropriate)
    - Integration of contraception follow-up into routine HIV follow-up appointments

2.2 Intervention implementation

After the intervention is fully designed, it will be implemented at twelve "intervention" FACES-supported PSC's using a model that will ultimately include integrating HIV and FP services in all 18 sites. Implementing the intervention will consist of:

- Training clinic staff in family planning counseling and method provision
- Introducing family planning assessments into all clinic visits via incorporating inquiries as to family planning needs on all medical forms, thereby prompting provider questioning
- Introducing new client counseling sessions and education materials into the PSC care infrastructure
- Devising a system of procuring, inventorying and dispensing contraceptive methods, including at the very least condoms, oral contraceptives and injectables. At sites where it is feasible, IUDs and implants will also be made available. When a method is not available at the site (such as in the case of surgical sterilization), clients will be referred to nearby centers that offer the method.

At both the intervention and control sites, a brief family planning module will be integrated into the standardized clinic encounter form that is used at each client visit. This module will prompt the clinician to ask if the client is currently using contraception and his or her fertility intentions. Simply put, if the client is using a method, the provider will be ask if he or she is interested in changing the method; if the client is not using a method and does not desire pregnancy, the provider will be ask if he or she is interested in starting a method.

- At "intervention" sites clients will then be extensively counseled about contraception and will be provided any needed contraceptive methods on site.

- At "control" sites, clients will be provided with contraceptive services through the centers' standard mechanisms, which generally involve referral to an affiliated family planning program that may or may not be on-site.

Prior to implementation of our intervention, we will resubmit the protocol for review at SSC/ERC for review of the final design of any educational materials developed.

3. Part III: Post-intervention data collection (Months 7-15)

3.1. Post-intervention site-based data collection

Over the following nine months, data on contraceptive prevalence, contraceptive methods used and pregnancy (including degree to which pregnancy was planned and desired) will be collected, as part of routine care, at the 18 sites (twelve intervention and six control sites) among women age 18-45 and men age 18 and older. Data will also be collected on whether contraceptive counseling was performed at each clinic visit. This information will be collected in the standardized clinic encounter form from all HIV-positive women and men at the time of each visit to the site. At each of the sites, a random sample of 10-15% of all charts will be reviewed during the post-intervention period to determine if the family planning module of the clinical encounter form is being filled out. A draft of the questions that will be used to collect the site-based data is included in Appendix A.

3.2. End-of-study KAP survey of female and male clients

In the final three months of the study, a convenience sample of approximately 28 female clients and 28 male clients will be recruited from each of 18 sites (N=488 female and 488

male patients). All 976 participants will complete the family planning KAP survey that was used at baseline with additional questions about the acceptability of family planning services offered at the site. Note that some of these participants may have also participated in the baseline KAP survey. The final version of the survey will be submitted to the IRB prior to initiating data collection.

The survey will be administered face-to-face by an interviewer in the respondent's preferred language in a private area of the centre. Inclusion criteria for the client KAP survey will be: 1) HIV-positive woman or man obtaining care at the FACES site, 2) not currently pregnant (if female), 3) age 18 to 45 years old if female and age 18+ if male; and 4) willing and able to give informed consent. Participants will be excluded if they do not meet the inclusion criteria listed above. Participants will be reimbursed a maximum of USD$3 (approximately 200 Kenyan Shillings at current exchange rate) for transport.

3.3. End-of-study survey with clinic staff at sites

In the final month of the study, 30-40 clinic providers will be contacted and will be administered a follow-up survey. We will attempt to interview the same providers who participated in the basesline interview, but due to staff turnover, this may not be possible. The questionnaire will cover the same topics as the baseline survey with additional questions examining the feasibility of offering family planning services in their site during the study period. The survey will include several open-ended questions that will be tape recorded (if the participant gives his/her consent), transcribed, translated to English (if interview not performed in English), and a content analysis will be performed using the qualitative software ATLAS.ti. Clinic staff will be reimbursed with a book voucher worth approximately USD$5 (approximately 350 Kenyan Shillings at current exchange rate) for participating in the survey. The final version of the survey will be submitted to the IRB prior to initiating data collection.

In order to further assess the feasibility of integration of family planning into HIV care and treatment program, we also plan to collect data on the cost of setting up and providing integrated services compared to the referral-based services. Collection of the cost data will involve meetings, document review, and interviews with staff in each of the study clinics. Specifically, data collection will take place via (1) review of service registers in intervention and control settings, (2) review of locally- and centrally-stored financial records, (3) intervention and control clinic visits to confirm resource use and allocation findings, and (4) semi-structured interviews with key administrative, clinical and financial staff. Where possible, cost and output data collected at the facility level will be also be verified against centralized accounting systems. No identifiable patient-level data will be collected, and staff will not be reimbursed for participating in costing interview.

3.4. End-of-study open-ended interviews with clients

In the final month of the study, open-ended interviews will be performed with 30 female and 30 male patients recruited from the 18 sites. The purpose of these in-depth interviews will be to obtain qualitative information about their experience undergoing contraceptive counseling and receiving family planning methods at the site and the acceptability of these services. The final version of the interview guide will be submitted to the IRB prior to initiating data collection.

A convenience sample of 30 women and 30 men will be invited to participate in the open-ended interview. This interview will be performed in the respondent's preferred language in a private area of the centre and will take approximately 30 minutes to complete. The inclusion criteria will be:

1) HIV-positive woman or man obtaining care at the PSC site
2) Not currently pregnant (if woman)
3) Age 18 to 45 years old if female; age 18+ if male
4) Has not received vasectomy or bitubal ligation
5) Willing and able to give informed consent

All interviews will be tape recorded (if the client gives his or her consent), transcribed, translated to English, and a content analysis will be performed using the qualitative software ATLAS.ti. Subjects will be reimbursed a maximum of USD$3 (approximately 200 Kenyan Shillings at current exchange rate) for transport.

Study and data management

The proposed study will utilize administrative offices at Suba District Hospital and/or Migori District Hospital. The study will maintain space for a desk, computer, and a locking file cabinet. The 18 study sites will be clinics within Suba, Kisumu East, Rongo and Migori District MOH. The study staff will include a study coordinator and a team of local language interviewers/research assistants. The study coordinator, under investigator guidance, will supervise the data collection, assist in intervention design, oversee the intervention implementation, and conduct regular site visits. He or she will also be responsible for data checking, quality control, and overall management of the data. Local language interviewers /research assistants will be responsible for abstraction of data from patient records and for conducting all interviews with clients and providers.

Digital recordings of the open-ended interviews with clients and clinic staff will be uploaded to a computer and transcribed into MS Word files by staff experienced in transcription, who will not have access to any identifying information on the participants. Recording and transcription files will be password-protected. Data from the surveys performed with female clients and clinic staff will be entered into SPSS by a member of the research team. These files will also be password-protected.

ENDPOINTS

Primary Outcomes (data abstracted from clinic encounter forms from all HIV-positive clients):

1. Contraceptive prevalence
   a. Will compare post-intervention data from intervention sites to data collected over same time period from control sites
2. Pregnancy rate (number of pregnancies/number of unique clients seen during post-intervention data collection period) and percent of pregnancies that are unplanned
   a. Will compare post-intervention data from intervention sites to data collected over same time period from control sites

Secondary Outcomes (data collected from KAP surveys and in-depth interviews):

1. Knowledge of contraceptive methods among HIV-positive clients
   a. Will compare pre-intervention rates from baseline data to post-intervention data at intervention sites

    b. Will compare pre-intervention rates from baseline data to post-intervention data at control sites
    c. Will compare post-intervention data from intervention sites to data collected over same time period from control sites
2. Knowledge of contraceptive methods among providers, including peer counselors, at HIV care and treatment centers
    a. Will compare pre-intervention rates from baseline data to post-intervention data at intervention sites
    b. Will compare pre-intervention rates from baseline data to post-intervention data at control sites
    c. Will compare post-intervention data from intervention sites to data collected over same time period from control sites
3. Acceptability of family planning services
    a. Will compare pre-intervention rates from baseline data to post-intervention data at intervention sites
    b. Will compare pre-intervention rates from baseline data to post-intervention data at control sites
    c. Will compare post-intervention data from intervention sites to data collected over same time period from control sites
4. Feasibility of providing family planning services at HIV care and treatment centers (data from post-intervention surveys of clinic staff)
5. Reproductive intentions of HIV-infected clients receiving care and treatment (data from baseline client surveys and in-depth interviews)

STATISTICAL/ANALYSIS PLAN

Sample size considerations

Data abstracted from clinical encounter forms. We have used ACLUSTER program to generate the number of PSC pairs (or clusters) that we will need to assess changes in contraceptive prevalence and unintended pregnancy. The first input into this program is the size of each cluster (or the number of eligible subjects available at each PSC site). There are a total of 36 FACES-supported PSC sites in Migori, Rongo, Kisumu East and Suba Districts, which are not all equal in size. The first calculation takes into account how we determine the average number of non-pregnant females of reproductive age that are potentially recruitable at each site. The other main input is the desired effect size we aim to be able to measure. Additional inputs include alpha, power and the Intra-cluster Correlation Coefficient (a measure of how similar we expect the two sites in each paired cluster to be to each other, for which we selected an intermediate value). Based on this we need 18 sites, 12 with the intervention and 6 control to be able to evaluate the integration model we propose as described in more detail below.

For the main cluster-randomized trial, the following data were inputted into the ACLUSTER program to generate an estimated number of clusters needed:

1. Number of subjects per cluster or site
    a. The eligible sample includes all FACES-supported PSCs in Suba, Kisumu East, Rongo and Migori Districts. The enrollment in December 2007 at the 16 FACES-supported sites in Suba District was 10,280, at the four sites in Kisumu was 6,593, at the five sites in Rongo was 1,338, and at the 11 FACES-supported sites in Migori District is 4,882.

   b. Because there are not accurate data about the number of non-pregnant women of reproductive age at these sites, we applied estimates based on observations of client demographics at the FACES site in Kisumu, where 65% of all clients are adult females, 89% of the female population is of reproductive age, and 90% of the reproductive age females are not pregnant.
   c. Applying these estimates, our eligible sample is approximately 12,023 women at 36 sites, an average of 333 women per site. For the sample size calculation, a conservative estimate of approximately 200 eligible women per site was used to allow for inclusion of smaller sites.
2. Effect size on primary outcome.
   a. The primary outcome upon which we based our sample size calculation is contraceptive prevalence. According to the 2003 Kenya Demographic and Health Survey, approximately 22% of women in a union in Nyanza Province use a modern contraceptive method. This level of contraceptive prevalence is similar to that seen among clients at the FACES sites. We would like to be able to detect a 8% rise in contraceptive prevalence to 30%.
3. Assumptions
   a. Two-sided alpha of 0.05
   b. Power of 0.8
   c. Intra-cluster Correlation Coefficient of 0.01
4. Sample size.
   a. Using these estimates, the needed sample size is approximately 140 women at each of the 18 sites, for a total sample of 2,520 women.
   b. We plan to initially recruit a similar number, 2,520, of male clients, however our primary outcome for men will be the proportion of male clients who report they or their partner are interested in starting a new FP method after receiving FP counseling during their visit. In order to accurately calculate this sample size, baseline data is needed to estimate the baseline proportion of male clients who would express interest in starting a new FP method. Thus, after the baseline data collection phase a more accurate sample size for male clients will be calculated.

KAP surveys. Because the largest sample sizes are required for expected proportions nearer to 0.5 than to 0 or 1, we will take the worst-case scenario and assume that for each individual question on the survey approximately half of the participants will answer correctly. We hypothesize that people in the intervention sites will be more knowledgeable after the intervention at the end of the study, so we would like to test for at least a ten percentage point difference in correct answers among patients in the intervention sites (~60% correct) compared to patients in the comparison sites (~50% correct), as well as a 10% increase in correct answers among patients in the intervention sites compared to the baseline results. For a one-sided test of two proportions using two samples, power of 0.8, and an alpha level of 0.05, the required sample size is 325 patients from the intervention sites and 163 patients from the comparison sites for both men (N=488) and women (N=488).

Open-ended interviews. A convenience sample of 30 women and 30 men will be invited to participate in the open-ended interview. The sample size for this qualitative component of the study is based on a reasonable assumption of the number of interviews needed to achieve saturation in the variety of responses obtained.

Data analysis plan

Analyses of data from the study health facilities, given the randomized cluster design, will mainly be conducted at the cluster (health facility) level. Individual level analyses will take into account

cluster effects. Primary analyses will be based on the “intention to treat” (ITT) principle, with facilities randomized to the intervention group classified as delivering “integrated services” regardless of whether or not individual women actually received integrated services.

Preliminary analyses will be conducted to compute study outcome variables and to summarize characteristics of control and treatment sites to assess the effectiveness of randomization. Initial analyses also will involve inspection of the frequency distributions of variables to identify outlying or unusual values. Following univariate descriptions of the data, bivariate and multivariate associations will be assessed as described below.

Analysis of the primary outcomes, contraceptive prevalence and unintended pregnancy rates, and the secondary outcomes, acceptability of services and knowledge related to contraception, will be based on a comparison of means or proportions between study arms using a weighted t-test that accounts for correlations between individual outcomes within sites. Even though there are only 12 health facilities in the intervention arm and 6 health facilities in the comparison arm, each mean or percentage will be based on a relatively large number of women, and the normal approximation to the binomial distribution can be used. These tests will be evaluated based on a two-sided alternative hypothesis, 80% power, and a 5% type I error rate. The same basic approach will be used for the other primary and secondary outcomes. Results will be summarized as odds ratios for models based on binary responses (e.g. use of contraception or experiencing unintended pregnancy), and mean changes for continuous responses (e.g. score on knowledge test).

As noted above, All open-ended interviews will be tape recorded (if the woman gives informed consent), transcribed, translated to English. A content analysis will be performed using the qualitative software ATLAS.ti in order to describe themes in women’s experiences undergoing contraceptive counseling, receiving family planning methods and in the acceptability of these services.

The ACLUSTER software program (Version 2.0) for the Design and Analysis of Cluster Randomized Trials developed by UNDP/UNFPA/WHO/ World Bank Special Programme of Research, Development and Training in Human Reproduction of the World Health Organization, as well as the SPSS statistical software package will be used for analyses. For qualitative data, the ATLAS.Ti software program will be used to code and perform content analysis of the open-ended interviews. A detailed analysis plan will be prepared in the beginning of the study period.

TIME FRAME

Study Timeline:

	Dec-’07-Jan ‘09	Feb-Mar ‘09	Apr-May ‘09	Jun-Jul ‘09	Aug-Sep ‘09	Oct-Nov ‘09
Conduct baseline site-based data collection	X	X				
Conduct baseline acceptability questionnaires and interviews with female and male clients	X	X				
Finalize design of family planning integration model	X	X				
Conduct baseline interviews with	X					

providers on knowledge, attitudes and practices related to family planning						
Train clinicians and community health workers in integrated model		X	X			
Implement the intervention at all sites		X	X			
Post-integration data collection at all sites on pregnancy status and contraceptive use			X	X	X	X
Conduct follow-up acceptability questionnaires and interviews with female and male clients				X	X	
Interviews with providers on acceptability and feasibility				X	X	
Data analysis						X
Dissemination of findings						X

ETHICAL CONSIDERATIONS

The study protocol and forms will be submitted to the UCSF Committee on Human Research as well as the Centre Steering Committee, Scientific Steering Committee, and Ethical Review Board of KEMRI, the Kenya Medical Research Institute.

Human Subjects Involvement and Characteristics: The human subjects who will be involved in the proposed study will be HIV-positive women and men, as well as clinic staff, at the study health facilities in Suba, Kisumu East, Rongo and Migori Districts, Nyanza Province, Kenya. Clients of the FACES-supported clinics and clinic staff will be invited to participate in face-to-face surveys and interviews, each of which will take 30-45 minutes to complete. In addition, data will be abstracted from the patient records to measure contraceptive prevalence and pregnancy rates. It is necessary to include HIV-positive women and men in this study as they are the main beneficiaries of integrated family planning and HIV services and the effectiveness of this approach can only be appropriately judged based on their experiences and outcomes with integrated vs. non-integrated services. The research will be performed at health facilities in Suba, Kisumu East, Rongo and Migori Districts, Nyanza Province, Kenya, as a collaboration between colleagues from the Kenya Medical Research Institute (KEMRI), Family AIDS Care and Education Services (FACES) and UCSF and Ibis Reproductive Health. The collaborating institutions will be equally involved and support implementing the integrated or non-integrated services at the study health facilities, abstraction of data from patient records, developing the interview guides and informed consent forms, as well as participating in analysis, interpretation, and dissemination of the study findings.

Sources of Materials: Data on contraceptive prevalence and pregnancy rates will be abstracted from information normally obtained in patient records as a part of their regular HIV care. The form used to abstract the data will not include the woman's name or address. The form will include a patient study ID number and the client's medical record number in order to be able to track treatment and outcomes from her medical record over time. However, medical record numbers will not be entered into the study database, only the study ID numbers.

The baseline surveys with female and male clients will be filled out during a face-to-face interview after obtaining informed consent (see below). No identifying information will be

collected as part of the surveys, and the questionnaires will be labeled only with a study ID number that is not linked to the client's medical record.

For the open-ended interviews with clients and clinic staff, after obtaining permission from participants through an informed consent process (see below), the interviews will be audio-recorded. The recording will not be initiated until after the informed consent process is complete and any initial introductions that might include identifying information have been completed. The recording will not include the participant's name or any identifying information, only a study ID #. A separate form will be used to record information on the participant's socio-demographic characteristics (age, education, marital status, number of living children, employment, etc.) but will not include the participant's name, birth date, or specific identifying information, only the study ID #. Audio recordings will be transcribed (and translated into English if necessary) by persons who do not have access to identifying information on the participants.

Potential Risks: Potential risks to clients participating in the study include any social risks involved if information they reveal about their HIV status or other sensitive issues were to be disclosed outside of the research. Potential risks to clinic staff participating in the interviews include the above mentioned social risks, as well as a potential employment risk if negative information they reveal about their work environment or work practices were to be disclosed to their supervisors. Potential interview participants (both clients and clinics staff) will have the option of not participating in any part or the full interview, or refusing the audio-recording, without any adverse consequences for their medical treatment or employment.

Informed Consent:

Baseline and follow-up surveys with male and female clients
Male and female clients will be invited to participate in the surveys (both baseline and follow-up) after they have completed their visit at the PSC site. They will be given information about the study and their rights as a part of the informed consent process. If they agree to participate, they will be asked to sign an informed consent form. As part of this informed consent process, potential participants will be informed of: (1) the purpose and methods of the study, (2) procedures to protect the confidentiality, (3) their rights to withdraw from the study at any time, (4) the fact that their participation or non-participation will not affect the medical care that they receive, and (5) persons to contact if they have any questions about the study after the completion of the interview. The participating clients will also be given a copy of the consent form to keep. This page will include names and phone numbers of persons to contact with any questions regarding the study. The consent form for clients participating in the surveys is included in Appendix C. The interviewer will also ask follow-up questions to ensure that the informed consent process has been well understood by the potential participant.

Baseline and follow-up open-ended interviews with female and male clients
A portion of clients who participate in the survey will be invited to participate in an additional open-ended interview. Before beginning the interview, informed consent will be obtained for the interview. The informed consent process for the interview will be identical to that of the survey with the addition of requesting permission to record the interview. If they refuse, audio recording will not be used; the interviewer will then take detailed notes during the interview. The consent form to be used with clients undergoing the open-ended interview is included in Appendix D.

Baseline and follow-up interviews with clinic staff
Health care workers in study facilities will be approached by the interviewer at a convenient time, either during their lunch break or before or after office hours. They will be given

information about the study and their rights as a part of the informed consent process, and if they agree to participate, they will be asked to sign an informed consent form. This consent form will explain that they will be asked to participate in a one-time face-to-face interview. As part of this informed consent process, potential participants will be informed of: (1) the purpose and methods of the study, (2) procedures to protect the confidentiality, (3) their rights to withdraw from the study at any time, (4) the fact that their participation or non-participation will not affect their employment at the study health facilities, and (5) persons to contact if they have any questions about the study after the completion of the interview. They will also be asked for permission to record the open-ended portion of the interview. If they refuse, audio recording will not be used; the interviewer will then take detailed notes during the interview. The participating clinic staff will also be given a copy of the consent form to keep. This page will include names and phone numbers of persons to contact with any questions regarding the study. The interviewer will also ask follow-up questions to ensure that the informed consent process has been well understood by the potential participant. The consent form to be used with clinic staff in this study is included in Appendix E. Clinic staff that are interviewed to collect cost data will not be asked to sign a consent form as this is standard for costing analyses.

Baseline and post-intervention site-based data collection
Informed consent will be obtained from female clients age 18-45 and male clients age 18 and above to obtain permission to abstract data from their medical records at the FACES site. They will be given information about the study and their rights as a part of the informed consent process. If they agree to allow data abstraction from their medical record, they will be asked to sign an informed consent form. As part of this informed consent process, potential participants will be informed of: (1) the purpose and methods of the study, (2) procedures to protect the confidentiality, (3) their rights to withdraw from the study at any time, (4) the fact that their participation or non-participation will not affect the medical care that they receive, and (5) persons to contact if they have any questions about the study after the completion of the interview. The participating clients will also be given a copy of the consent form to keep. This page will include names and phone numbers of persons to contact with any questions regarding the study. The consent form for clients participating in the site-based data collection is included in Appendix F. The interviewer will also ask follow-up questions to ensure that the informed consent process has been well understood by the potential participant.

Protection against risk: Forms for abstraction of data on the care and outcomes of HIV-positive clients will not include names or addresses, only the client's medical record number and his or her study ID number. These forms will be kept in a locked cabinet at the research office in Suba District and no one outside of the core research team members will have access to these forms.

All interviews with clients and clinic staff will be conducted in a private room designated for this purpose at the study health facilities. No identifying information will be included on the audio recording of the interview. The form for recording socio-demographic characteristics of participants will not include the participant's name or other identifying information. Interview recordings, transcripts, and forms will be stored in a locked cabinet at the research office in Suba District and on a computer only accessible by core members of the research team. When these procedures are followed, it is highly unlikely that any of the information revealed by participants during the course of the interviews will be disclosed to anyone outside the research team. Given the small number of health care providers at some facilities, results will never be presented separately by facility, as that might identify specific participants. Results will only be presented by site type (sub-district hospital vs. dispensary) or by study group (integrated vs. non-integrated).

Compensation
Participants will receive a monetary reimbursement for participation in each survey or interview to cover transportation to and from the site and for time spent in the study. No reimbursement will be given to clients who consent to data abstraction from their medical record. Reimbursement amounts will be a maximum of USD$3 (approximately 200 Kenyan Shillings at current exchange rate) for clients who participate in the survey or open-ended interview, and will be a book voucher worth approximately USD$5 (approximately 350 Kenyan Shillings at current exchange rate) for the clinic staff who participate in the interview. Clients who contribute site-based data that is collected on the standard clinic encounter forms will not be reimbursed since no procedures unique to the study will be done and they will receive standard care at the sites.

Potential benefits of the proposed research to the subjects and others
The intervention clinic sites will provide family planning services, including immediate contraceptive method provision. Patients at these intervention sites will receive contraceptive counseling and services as part of their regular care, these services are not currently part of the standard HIV care. Patients at the control sites will receive standard of care family planning services which generally involves referral elsewhere. All patients will receive counseling about barrier protection. All subjects will receive the satisfaction of knowing that participation in this research will help people in their communities and elsewhere gain increased access to reproductive health and choice in the future.

EXPECTED APPLICATION OF RESULTS
If integration of family planning services into HIV care and treatment appears feasible, acceptable, and effective at decreasing unintended pregnancies among HIV-infected women, the family planning intervention will be introduced into all FACES-supported PSC clinics. If the intervention does not appear to be feasible, acceptable, or effective based on the results of the trial, assessment of the integration will be conducted to try to identify ways in which the family planning intervention could be improved or changed. Modification of the intervention and reintroduction of it into HIV care and treatment will be considered based on trial results. Ultimately, the findings of this research will help guide future research, policy, and interventions in the area of integrating family planning into HIV care. The ultimate goal is to create a feasible, acceptable, and effective family planning intervention that can be modified and applied elsewhere in Kenya and throughout Sub-Saharan Africa in order to increase access to effective contraception among HIV-infected women, thereby allowing them to make reproductive choices and to decrease unintended pregnancies.

LIMITATIONS

Potential Design Pitfalls
Non comparable sites: Government facilities in Suba, Kisumu East, Rongo and Migori Districts provide varying levels of care from basic outpatient dispensaries with minimal staff and facilities and services, to the district hospital with comprehensive diagnostic and treatment services including inpatient and outpatient facilities. Comparisons of facilities offering different levels of care would likely introduce confounding factors into the study outcomes. To avoid this potential confounding influence, we will perform an initial assessment of each of the possible sites and divide them into similar groups according to the number of clients served, the type of facility, the geographical location, and the type of HIV and family planning services currently available on site. We will group the sites into two strata based on this information; six intervention and three

control sites will be selected from each of the two strata. In addition, intra-cluster correlation is factored into our data analysis plan (see above section on statistical/analysis plan).

Non-uniform protocol implementation: We will train all health staff and paramedical staff in the standard operating procedure for each HIV care and treatment clinic, including the standard operating procedures for integration or non-integration of family planning services with HIV care and treatment. However, as in all settings, it is impossible to control for individual staff motivation. Therefore, it is possible that some sites will follow the protocol more stringently than others and that some sites will recruit patients more enthusiastically than others. With a randomized design the potential confounder of staff motivation should be evenly distributed in both the intervention and the control arms, thereby minimizing the problem of confounding influences.

Mixing among sites: Currently, FACES staff report that it is uncommon for clients to receive services from more than one site. However, it is possible that a woman who usually attends a site that is randomized to be a control site will at some point obtain care from an intervention site and obtain integrated family planning services. If she then returns to the control site, she will contribute follow-up data to that site. We will attempt to collect data about obtaining care at other sites, but we anticipate that such mixing will be minimal, especially because the intervention and control sites that will be selected are not near one another.

Additional limitations
An additional limitation of the study is its generalizability: while the results will be representative of the setting in Suba, Kisumu East, Rongo and Migori Districts in Nyanza Province, it is unclear how generalizable they will be to other regions of Kenya or Sub-Saharan Africa. Nonetheless, combined with information about the study setting, the results will be useful to policy makers and program planners throughout the region as they develop services to improve the reproductive health of HIV-infected women.

INVESTIGATOR ROLES AND RESPONSIBILITIES

Craig Cohen, M.D., MPH; Director of FACES, Principal Investigator: Dr. Cohen is an Associate Adjunct Professor in the Department of Obstetrics, Gynecology and Reproductive Sciences at UCSF. He conducts research in two major areas: female reproductive tract infections and HIV/AIDS care and support in developing countries. For nine years, based at the University of Washington, he established a research program in Nairobi, Kenya, in conjunction with the University of Nairobi and the Kenya Medical Research Institute. Dr. Cohen has been the Principal Investigator (PI) for HIV care and support investigations supported by World Health Organization and the Rockefeller Foundation and he is Director of the CDC PEPFAR-funded Kenya-based FACES HIV/AIDS care and support program. Dr. Cohen's role in this study is to facilitate the interaction between FACES, KEMRI and the research team; plan study initiation; and oversee study progress.

Elizabeth Bukusi, MB.ChB, M.Med, MPH; KEMRI Co-Director, FACES Program Director, Co-Principal Investigator: Dr. Bukusi is a Principal Research Officer and Co-Director of the Research Care and Training Program (RCTP) at the Center for Microbiology Research, KEMRI and an Honorary Lecturer at the University of Nairobi. Dr. Bukusi received her MB.ChB. and her M.Med. in Obstetrics and Gynecology from the University of Nairobi and she earned her MPH and PhD at the University of Washington. Dr. Bukusi has been site PI on several HIV and reproductive health investigations in Kenya, including "Male microbicides genital hygiene and

HIV risk behavior" and "Phase III Randomized Placebo Controlled Trial of HSV-2 Suppression to Prevent HIV Transmission Among HIV-Discordant Couples." Dr. Bukusi's role in this study is to assist the PI with study initiation and to oversee study progress.

Daniel Grossman, M.D.; Senior Associate, Ibis Reproductive Health, Co-Principal Investigator: Dr. Grossman received his Bachelor's of Science in Molecular Biophysics and Biochemistry from Yale University and an M.D. from Stanford University. He completed his residency in Obstetrics and Gynecology at the University of California, San Francisco (UCSF), where he currently maintains a clinical faculty appointment. Dr. Grossman has been the PI or co-PI of several studies related to contraception, fertility and prevention of sexually transmitted infections in both the United States and Latin America. Dr. Grossman's role in the study is to assist the PI with study design and implementation and to oversee study progress.

Sara Newmann, M.D., MPH; Fellow in Family Planning, University of California, San Francisco, Co-Investigator: Dr. Newmann is an assistant clinical professor in the Department of Obstetrics, Gynecology and Reproductive Sciences at UCSF at the University of California, San Francisco (UCSF). She received her M.D. from Brown University and her MPH from Harvard University. She completed her residency in Obstetrics and Gynecology at Harvard University and remained on faculty at Harvard University for two years. She then completed a two-year fellowship in family planning at UCSF where she is currently on faculty. Her interests include facilitating access to family planning for women in vulnerable populations. She has experience working in family planning and HIV prevention in Indonesia, the United States, El Salvador, and India. Dr. Newmann's role in the study will be to assist Drs. Cohen, Grossman, and Bukusi with execution of the study plan. She will assist with all aspects of the study including hiring and training study staff, collection of baseline data, study and intervention design, implementation of the intervention, collection of quantitative and qualitative data, and data analysis and dispersion.

Starley Shade, PhD; Assistant Adjunct Professor, University of California, San Francisco, Co-Investigator: Dr. Shade is an epidemiologist and biostatistician with extensive experience in the design, conduct and analysis of randomized trials and evaluation research in clinical settings. For eight years, she served as the statistician for the Positive Health Program at UCSF, where she participated in the design, data management and analysis of many clinical trials including those conducted by the CPCRA network. Since completing her PhD at UC Berkeley in 2006, her work has focused on developing and improving quantitative methods, including electronic patient monitoring, in the evaluation of clinic- and community-level research. Over the past two years, she has participated in the development and implementation of the OpenMRS patient monitoring system used by FACES and currently leads two evaluations of patient monitoring systems in Kenya and Mozambique. Dr. Shade will oversee the abstraction of data from clinical encounters and data management and analysis of all quantitative study data.

Katie Doolan, MPH; FACES Research Coordinator, University of California, San Francisco, Co-Investigator: Ms. Doolan received her MPH from the University of Cape Town, South Africa and currently manages the research studies that fall within Migori District, Kenya. Ms. Doolan's role in the study will be to assist the Investigator's in overall study coordination from UCSF and Kenya, including liaising between KEMRI and UCSF study teams, assisting with data collection at baseline and post-intervention and dispersion of results.

Training benefits

Training benefits of this project are related to study methodology and analysis. All study staff will receive training related to study methods and research with human subjects, skills that will be useful in future research. The statistician will receive specific training and support related to qualitative analysis. It is likely that researchers-in-training from KEMRI and UCSF, including medical students, residents, graduate students and fellows will become involved in this study and receive specific training in new skills.

BUDGET

Note that all amounts are in US dollars.

BUDGET FOR INITIAL BUDGET PERIOD<br>Integrating FP into HIV Care and Treatment					FROM<br>11/1/2008	THROUGH<br>10/31/2009
PERSONNEL *(Applicant organization only)*					DOLLAR AMOUNT RE *(omit cents)*	
	ROLE	Percent Effort	Salary	Benefits	SALARY & BENEFITS REQUESTED	TOTAL
Craig Cohen	Principal Investigator	5%	11,118	1,890	13,008	13,008
Dan Grossman	Co-Investigator	10%	20,150	3,426	23,576	23,576
Sara Newman	Co-Investigator	10%	15,495	2,634	18,129	18,129
Katie Doolan	Project Manager	30%	17,531	4,383	21,914	21,914
TBA	Post Award Analyst	5%	2,569	642	3,211	3,211
Starley Shade	Statistician	5%	4,733	947	5,680	5,680
**SUBTOTALS**			→		85,518	85,518
SUPPLIES *(Itemize by category)*						
Computer and supplies	3,000					3,000
TRAVEL						
UCSF International Travel	11,424					11,424
OTHER EXPENSES *(Itemize by category)*						
UCSF Rent	5,246					
UCSF IT	462					5,709
KEMRI DIRECT COSTS						139,461
**SUBTOTAL DIRECT COSTS**						245,112
KEMRI FACILITIES AND ADMINISTRATIVE COSTS (15%)						20,919
UCSF FACILITIES AND ADMINISTRATIVE COSTS (26%)						33,969
**TOTAL COSTS FOR INITIAL BUDGET PERIOD**						**$300,000**

KEMRI SUBCONTRACT BUDGET						
**NAME**	**DESIGNATION**	**EFT**	**Annual Salary**	**Benefits @15%**	**TOTAL**	**TOTAL**
**Personnel**					**KSHS**	**US $**
Dr. Elizabeth Bukusi	Site PI	1%				1,500.00
TBE	FP Coordinator	100%	840,000.00	126,000.00	966,000.00	15,836.07
TBE	FP Ass. Coordinator	100%	480,000.00	72,000.00	552,000.00	9,049.18
TBE	Nurses	400%	1,680,000.00	252,000.00	1,932,000.00	31,672.13
TBE	Interviewers	300%	720,000.00	108,000.00	828,000.00	13,573.77
TBE	Data Clerks	300%	720,000.00	108,000.00	828,000.00	13,573.77
Barrack Onyango	Program Manager	10%	240,000.00	36,000.00	276,000.00	4,524.59
Dr. Rosemary Nguti	Data Analysis	5%	215,000.00	32,250.00	247,250.00	4,053.28
**Personnel Total**					**5,629,250.00**	**93,782.79**
**Travel**						
Local Travel						9,000.00
**Travel Total**						**9,000.00**
**Participant Costs**						
Participants Reimbursements						4,022.00
**Participant Costs Total**						**4,022.00**
**Others**						
Family Planning Training						10,000.00
Materials						8,000.00
Printing and Photocopying						3,000.00
Communication						3,642.00
Internet						1,080.00
Health Insurance						3934
Dissemination Meeting						3,000.00
**Others Total**						**32,656.00**
**KEMRI DIRECT**						**139,460.79**
KEMRI-INDIRECT-15%						20,919.1
**KEMRI TOTAL COSTS**						**160,380**

Budget justification

This project is supported by the Tides Africa Foundation.

UCSF Personnel (all staff receive benefits at 17% for academic staff and 25% for non-academic staff)

- Craig Cohen, MD, MPH, will serve as project PI and will be supported at 5%.
- Daniel Grossman, MD, will serve as Co-PI and will be supported at 10%.
- Sara Newmann, MD, MPH, will serve as Co-PI and will be supported at 10%.
- Katie Doolan, MPH will serve as the UCSF project manager and will be supported at 30%.
- Starley Shade, PhD, a biostatistician at UCSF, will be supported at 5%.
- To Be Assigned, Post Award Analyst at UCSF, will be supported at 5%.

UCSF Travel

Four round-trip tickets from San Francisco to Kisumu will be purchased at a total cost of $11,424..

Computers

Three laptops will be purchased for the Kenyan Family Planning staff at a total cost of $3,000.

Other Expenses

- $5,246 is requested in rent support for UCSF personnel located at 50 Beale Street in San Francisco.
- $462 is requested for IT support for personnel located at 50 Beale Street in San Francisco.

Indirect costs

An indirect rate of 26% is applied to all UCSF costs.

KEMRI

Personnel (all staff receive benefits at 15%)

- Elizabeth Bukusi will serve as Site PI and will be supported at 1% effort. Dr. Bukusi will give additional in-kind support of approximately 9%.
- A nurse/researcher will serve as the Family Planning coordinator to coordinate the project and will be supported full-time.
- An assistant coordinator also trained as a nurse will also be supported full-time.
- Four nurses who specialize in family planning will be supported full-time to mentor and assist clinicians with FP integration at the 18 sites.
- Three interviewers will hired to conduct interviews with clients and clinicians.
- Three data clerks will be supported full-time to perform data entry for the electronic medical records (OpenMRS system) and manage the data. An additional $3,000 is requested to support approximately 80% of an additional data clerk to perform data entry for the surveys and questionnaires detailed in the proposal.
- Barrack Onyango, the FACES Program Manager based at KEMRI, will be supported at 10% to perform administrative duties and supervise financial management.
- Dr. Rosemary Nguti, a statistician based at KEMRI, will be supported at 5% to assist with data analysis.
- Health insurance costs of $3,934 are requested to provide the twelve 100% EFT employees with health insurance. The current health insurance premium is 20,000 Ksh per annum per 100% EFT employee; this translates into $327.87 US dollars.

Travel

$9,000 is requested to support local travel throughout Nyanza Province over the course of the year-long project. To the extent possible, travel related to the project will be coordinated with other FACES activities to take advantage of program vehicles.

Participant reimbursements

- 640 HIV-infected clients will be interviewed, and each will be given $3 to compensate them for their time and travel.
- 30 clinicians will be interviewed, and each will be given a voucher worth $5 redeemable for a book.

Training and materials

- $18,000 is requested to support training activities and materials to be used in the clinic sites related to family planning. One 2-day training will be performed at a central location in Kisumu, another in Suba District, and another in Migori District (to which will also come staff from Rongo District). Travel costs for all participants will be supported. Additional follow-up trainings will be performed by the project coordinator at individual sites. Materials will include flip-charts, posters and pamphlets.
- $3,000 are requested to support an end-of-year dissemination meeting of findings to-date.

Additional costs

- Communication costs of $3,642 are requested to support project staff's cell phones and mobile data connection when needed.
- Internet costs of $1,080 are requested.
- Printing and copying costs of $3,000 are requested.

Indirect costs

An indirect rate of 15% is applied to all KEMRI costs.

REFERENCES

1. UNICEF. *Protection and support for orphans and families affected by HIV/AIDS*. HIV/AIDS and children [cited February 18, 2007]; Available from: http://www.unicef.org/aids/index_orphans.html.
2. *Prevention of HIV in infants and young children: review of evidence and WHO's activities*. 2002, World Health Organization: Geneva.
3. Duerr, A., et al., *Integrating family planning and prevention of mother-to-child HIV transmission in resource-limited settings.* Lancet, 2005. **366**(9481): p. 261-3.
4. *Linking Reproductive Health, Family Planning and HIV/AIDS in Africa*. 2006. UN Conference Center, Addis Ababa, Ethiopia.
5. Fleischman, J. and J.S. Morrison, *Integrating Reproductive Health and HIV/AIDS Programs. Strategic Opportunities for PEPFAR. A report of the CSIS Task Force on HIV/AIDS.* 2006, Center for Strategic and International Studies.
6. *Country Assessment: Kenya. Family Planning Needs in the Context of the HIV/AIDS Epidemic.* 2004, Family Health International; United States Agency for International Development.
7. Reynolds, H.W., M.J. Steiner, and W. Cates, Jr., *Contraception's proved potential to fight HIV.* Sex Transm Infect, 2005. **81**(2): p. 184-5.
8. *The Glion call to action on family planning and HIV/AIDS in women and children.* 2004, World Health Organization: Geneva.
9. Smart, T. (2006) *PEPFAR: Unexpected and unwanted pregnancies in women on ART highlights family planning gap*. Aidsmap news **Volume**,
10. Brocklehurst, P. and R. French, *The association between maternal HIV infection and perinatal outcome: a systematic review of the literature and meta-analysis.* Br J Obstet Gynaecol, 1998. **105**(8): p. 836-48.
11. *Kenya Demographic and Health Survey 2003*.???? in *MEASURE DHS STATcompiler*. 2003.
12. *Kenya Demographic and Health Survey 1998*. 1999, National Council for Population and Development; Central Bureau of Statistics (Kenya); Macro International: Kenya; Calverton, Maryland.
13. *Zambia Demographic and Health Survey 2001-2002*. 2003, Central Statistical Office (Zambia); Central Board of Health (Zambia); ORC Macro: Zambia; Calverton, Maryland.
14. *Family Planning Worldwide. 2002 Data Sheet.* 2002, Population Reference Bureau: Washington DC.
15. *National AIDS and STI control programme of the ministry of health (Kenya),* in *AIDS in Kenya*. 2005: Nairobi, Kenya.
16. Asiimwe, *Uganda TOO Final Report.*
17. Ooko, H., *FACES data regarding contraception use in Lumumba* 2007. p. email, Excel spreadsheet.
18. *Family Health International. Case Studies: Uganda, Cambodia.*, in *Network 2004*. 2004. p. 16-17.
19. King, R., et al., *A family planning intervention to reduce vertical transmission of HIV in Rwanda.* AIDS, 1995. **9**((suppl 1)): p. S45-S51.
20. Peck, R., et al., *The Feasibility, Demand, and Effect of Integrating Primary Care Services with HIV Voluntary Counseling and Testing. Evaluation of a 15-Year experience in Haiti, 1985-2000.* Journal of Acquired Immune Deficiency Syndromes, 2003. **33**: p. 470-475.

21. Sweat, M., *Cost effectiveness of nevirapine to prevent mother to child transmission in 8 African Countries.* AIDS, 2004. **18**: p. 1661071.
22. Stover, J., *Adding family planning to PMTCT sites increases the benefits of PMTCT*, in *USAID Issues Brief*. 2003, Bureau for Global Health.
23. Reynolds, H., *The Value of Contraception to prevent Perinatal HIV Transmission.* Sexually Transmitted Infections, 2006. **33**(6): p. 350-356.
24. Koh, K., *Suggestions for improving reproductive health care at FACES Clinic*. 2006, Columbia University: New York. p. 1-9.
25 WHO. *Hormonal Contraception and HIV: Science and Policy Africa Regional Meeting*. in *Hormonal Contraception and HIV: Science and Policy Africa Regional Meeting*. 2005. Nairobi.
26. Farrell BL, W.D., Kakande H, *Integrating Family Planning Counseling and Services into HIV Care and Treatment Services in Uganda:TASO/Mbale A Performance Needs Assessment*. 2006, The ACQUIRE Project, USAID: Kampala.
27. Kenyan Ministry of Health, D.o.R.H., *Family Planning Guidelines for Service Providers*. 2005, Nairobi.
28. Sanders R, H.K., Sheperd C, *Implementing integrated family planning and HIV / AIDS policies and programs: tools and resources*. 2006, Policy Project, Futures Group. p. 37.
